# Pentathiepins: A Novel Class of Glutathione Peroxidase 1 Inhibitors that Induce Oxidative Stress, Loss of Mitochondrial Membrane Potential and Apoptosis in Human Cancer Cells

**DOI:** 10.1002/cmdc.202000160

**Published:** 2020-05-06

**Authors:** Steven Behnisch‐Cornwell, Siva Sankar Murthy Bandaru, Martin Napierkowski, Lisa Wolff, Muhammad Zubair, Claudia Urbainsky, Christopher Lillig, Carola Schulzke, Patrick J. Bednarski

**Affiliations:** ^1^ Pharmazeutische/Medizinische Chemie Institut für Pharmazie Universität Greifswald 17489 Greifswald Germany; ^2^ Bioanorganische Chemie Institut für Biochemie Universität Greifswald 17489 Greifswald Germany; ^3^ Institut für Medizinische Biochemie und Molekulare Biologie Universitätsmedizin Universität Greifswald 17475 Greifswald Germany

**Keywords:** apoptosis, cancer cells, cytotoxicity, DNA fragmentation, glutathione peroxidase, oxidative stress, pentathiepin

## Abstract

A novel class of glutathione peroxidase 1 (GPx1) inhibitors, namely tri‐ and tetracyclic pentathiepins, has been identified that is approximately 15 times more potent than the most active known GPx1 inhibitor, mercaptosuccinic acid. Enzyme kinetic studies with bovine erythrocyte GPx1 indicate that pentathiepins reversibly inhibit oxidation of the substrate glutathione (GSH). Moreover, no inhibition of superoxide dismutase, catalase, thioredoxin reductase or glutathione reductase was observed at concentrations that effectively inhibit GPx1. As well as potent enzyme inhibitory activity, the pentathiepins show strong anticancer activity in various human cancer cell lines, with IC_50_ values in a low‐micromolar range. A representative tetracyclic pentathiepin causes the formation of reactive oxygen species in these cells, the fragmentation of nuclear DNA and induces apoptosis via the intrinsic pathway. Moreover, this pentathiepin leads to a rapid and strong loss of mitochondrial membrane potential in treated cancer cells. On the other hand, evidence for the induction of ferroptosis as a form of cell death was negative. These new findings show that pentathiepins possess interesting biological activities beyond those originally ascribed to these compounds.

## Graphical abstract

**Anticancer drug development**: Sulfur does it better! Pentathiepins inhibit glutathione peroxidase 1 (GPx1), cause a loss of mitochondrial membrane potential as well as oxidative stress in cancer cells, resulting in DNA strand breaks and inducing apoptosis. The loss of MMP appears to play a key role in their mechanism of action. Their qualities make pentathiepins interesting lead structures for anticancer drug development.

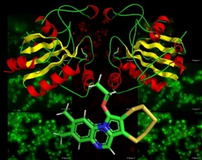

## Introduction

**Figure 1:**
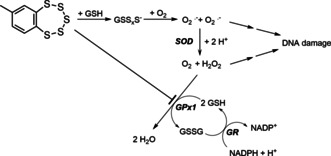
Postulated mechanism for the oxidative DNA damaging activity of 7‐methylbenzopentathiepin^16^, ^17^ and the role that GSH and GPx1 inhibition could play in this activity. GSH: reduced glutathione; GSSG: glutathione disulfide; GSS_x_S^−^: polysulfide anions; SOD: superoxide dismutase; GR: glutathione reductase.

The glutathione peroxidases (GPx) are critical antioxidative enzymes responsible for the intracellular destruction of H_2_O_2_ and organic peroxides. GPxs oxidize two glutathione (GSH) molecules to the glutathione disulfide (GSSG) while reducing H_2_O_2_ or the organic peroxide to either water or the corresponding alcohol, respectively. Eight isoenzymes of GPx are known, five of which (GPx1–GPx4 and GPx6) make use of an active site selenocysteine while for the other GPxs an active site cysteine is present.^1^ Under physiological conditions, the selenol (R−SeH) is ionized as a selenolate (R−Se^−^), which makes it very reactive towards oxidations by peroxides.^2^ A selenenic acid (R−Se−OH) is formed as an intermediate in the enzymatic degradation of the peroxide, which is recycled back to the selenolate by reaction with two equivalents of glutathione (GSH). One equivalent of peroxide produces a glutathione adduct in a substitution reaction, with a following reduction back to the selenolate with concurrent formation of the glutathione disulfide (GSSG; Figure 1). GSSG is restored to GSH by glutathione reductase (GR) under consumption of NADPH. The maximum velocity of the GPx‐reaction (*V*_max_) with the substrates GSH and H_2_O_2_ seems to be unlimited with increasing concentrations. The restoration of the enzyme is a reaction time limiting process and the saturation of peroxide degradation leads to the ping‐pong mechanism.^3^

The two most prevalent forms of glutathione peroxidase are GPx1, a cytosolic enzyme, and GPx4, which is located in cell membranes. GPx1 destroys water soluble peroxides whereas GPx4 degrades phospholipid peroxides in membranes. Interestingly, knockout mice of GPx1 show no special phenotype; they are fertile, have a healthy appearance and no special sensitivity against hyperoxia.^4^ On the other hand, mice with knockout GPx4 are not viable, presenting an intense phospholipid peroxidation in their cell membranes.^5^

In general, the antioxidative activity of GPx‐isoenzymes is associated with a positive effect for organisms. However, in the cases of cancer development and tumor progression, the role of the GPx is mixed. On the one hand, polymorphisms of GPx1 with low activity are known to cause enhanced oxidative stress accompanied by a higher risk in cancer progression for malignant lung, breast and prostate diseases.^6^ On the other hand, it is described that high GPx activity in malignant diseases can cause a poor prognosis.^7^ It has also been observed that increased GPx activity in cancer cells can be involved in the resistance mechanism against anti‐cancer drug treatment.^8^ Recently, GPx4 has been brought into contention as an antiferroptotic enzyme in the survival of therapy‐resistant cancer cells across diverse mesenchymal cell origins.^9^ Thus, the development of GPx inhibitors could offer a promising avenue to novel anticancer drugs.

Due to the shallow active site of GPx1 only a few inhibitors have been identified to date, all having relatively low specificity and sensitivity. The best characterized inhibitors of the GPx1 are mercaptosuccinic acid [HOOCCH(SH)CH_2_COOH, MSA] and other mercaptans,^10^ including tiopronin.^11^ Mass spectral evidence indicates that these thiols act as mechanism‐based inhibitors by first reacting with the selenenic acid in the enzyme active site to form a S−Se intermediate. A lysine residue next to the active site of the enzyme is then oxidized to a sulfonamide intermediate while the selenocysteine is restored to the selenol. The oxidation of a sulfenamide to a sulfonamide results in an irreversible inhibition of the enzyme.^11^ Gold and mercury compounds like auranofin, gold(I)thioglucose and methylmercury also have GPx1 inhibitory activity.^12^ The high effectivity of these compounds to GPx1 is explained by the avid affinity of gold and mercury to selenols, but this high affinity also minimizes selectivity; it is also known that these compounds act as potent inhibitors of the glutathione reductase and the thioredoxin reductase.^12^ More recently, we identified acylhydrazone heterocycles as weak inhibitors of the GPx1, with IC_50_ values greater than 100 μM.^8^ Various cell lines with resistance to cytotoxic agents, some of which showed upregulation of GPx1, were made sensitive again to the anti‐cancer drugs when co‐treated with these acylhydrazones.^8^

**Figure 2:**
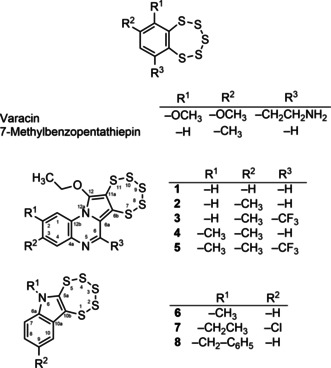
Structures and systematic numbering of pentathiepins.

Pentathiepins are a unique group of heterocyclic compounds with a chain of five sulfur atoms forming a stable cyclic heptagon annulated to an aromatic ring system.^13^ Some pentathiepins, such as varacin (Figure 2), are even natural products and have been identified in various ascidians of the genus *Lissoclinum*.^14^ They possess interesting activities against cancer cells, bacteria and fungi;^15^ for example, varacin has comparable cytotoxicity to that of doxorubicin.^16^ Simpler analogues of pentathiepins like 7‐methylbenzopentathiepine (Figure 2) also possess cytotoxicity in cancer cell lines.^17^ Early research indicated that one reason for their cytotoxicity could be their ability to cleave DNA via an oxidative type mechanism dependent on the presence of GSH^16^, ^17^ (Figure 1). This mechanism is generally accepted as an explanation for the cytotoxic activity of pentathiepins but evidence that this mechanism operates in cells is lacking.

In the current work we have synthesized eight in part novel pentathiepins possessing various heterocyclic rings (Figure 2) and evaluated them as potential GPx1 inhibitors. We report here the unexpected ability of the compounds to reversibly inhibit bovine erythrocyte GPx in sub‐micromolar concentrations. Moreover, the cytotoxic potential of the compounds to kill cancer cells *in vitro* as well as the mechanisms of cell death have been investigated. These results show that pentathiepins have more diverse effects on cellular systems than previously surmised.

## Results

### Chemistry

**Scheme 1:**
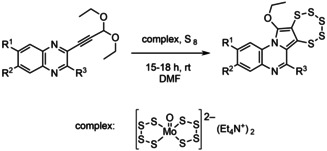
Synthesis of pentathiepins **1**–**5** by the molybdenum mediated diethoxy alkyne to pentathiepin route.

The syntheses of the two groups of pentathiepins (**1**–**5** and **6**–**8**) were achieved via two separate routes. The pyrrolo[1,2‐a]quinoxaline pentathiepins (**1**–**5**) were prepared according to the method of Zubair et al..^18^ The schematic synthetic protocol is depicted in the Scheme 1. The respective alkynyl quinoxaline precursors were synthesized on a multigram scale following the typical Sonogashira coupling reaction procedure.^18^ Originally it was attempted to synthesize molybdenum dithiolene complexes from the alkyne precursors by reaction with (Et_4_N)_2_ [*M*oO(S_4_)_2_]. However, the first thereby isolated product was surprisingly identified as a novel N‐heterocyclic pentathiepin.

**Figure 3:**
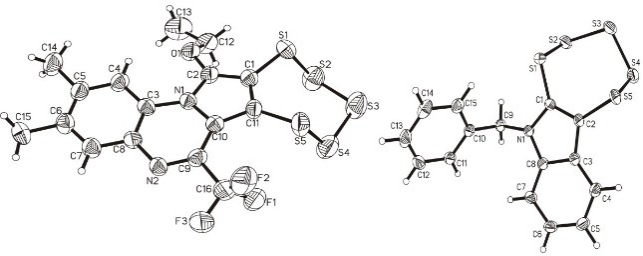
Molecular structures of **5** (left) and **8** (right), as determined by X‐ray structural analysis. Ellipsoids are drawn at the 50 % probability level.

As pentathiepins are biologically active molecules^13^ research in this field was continued leading to further quinoxaline based pentathiepin derivatives (**1**–**5**). In order to understand the role of the Mo^IV^ species, different alkyne precursors with and without ethoxy functional groups were tested under similar reaction conditions. As pointed out previously, the two ethoxy substituents of the precursors are required for the reaction to proceed according to the proposed reaction mechanism.^18^ Employing alkyne precursors without the diethoxy moiety results in the corresponding Mo^IV^ bis‐dithiolene complexes, instead of pentathiepins. This suggests that the Mo^IV^ complex plays a dual role: activating the triple bond and engaging in a redox reaction with elemental sulfur. Accordingly, S_8_ might be reduced to a S_5_^2−^ fragment while Mo^IV^ is oxidized to Mo^VI^ bound to the remaining S_3_ fragment. In course of the ongoing reaction elemental sulfur is formed, probably at least partly from the S_4_^−2^ ligands of the original complex, and released while re‐reducing molybdenum to the Mo^IV^ complex. After completion of the reaction, the resulting pentathiepin can be isolated as the major product from the reaction mixture. The remaining compounds constitute the molybdenum complex as well as side products (e. g., not yet identified Mo^IV^ derivatives and elemental sulfur). The molecular structure of derivative **5** was unambiguously established by X‐ray structural analysis albeit in comparably poor quality due to a twinning problem (Figure 3, left).

**Scheme 2:**
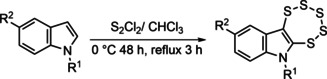
Synthesis of pentathiepins **6**–**8** by the disulfur dichloride route.

The indole‐based pentathiepins (**6**–**8**) were synthesized according to the method of Amelichev,^19^ whereby the respective indoles were reacted with S_2_Cl_2_ in CHCl_3_ in a one‐pot reaction (Scheme 2). An X‐ray crystal structure of the N‐benzyl derivate **8** confirmed the presence of the 7‐membered pentathiepin ring (Figure 3, right).

It has been reported that pentathiepins are instable in the presence of thiols such as GSH.^20^ Thus, the stability of a representative pentathiepin, **4**, in the presence of GSH was studied first by APCI‐MS. Incubations of **4** in the presence of 10 mm GSH, a biologically relevant concentration, were monitored at 23 °C for a period up to 1 h. No changes in the mass spectrum attributed to **4** were observed (Figure S31 in the Supporting Information), indicating good stability. Next, the stability of **4** was studied by RP‐HPLC in the same buffer and with the same GSH concentration (0.25 mM) used in the GPx assay (see below). Over the course of a 70 min incubation a nearly 30 % loss of the pentathiepin (Figure S32) was observed. However, since the GPx assay is only run for 20 min, the direct reaction between **4** and GSH would only account for a ca. 10 % loss of pentathiepin under assay conditions.

### Biology

#### Pentathiepins inhibit bovine and human GPx activity

**Figure 4:**
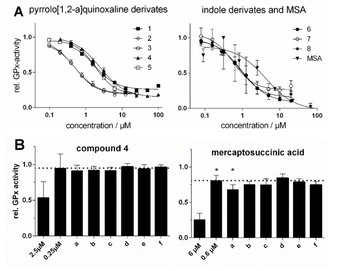
A) Concentration‐activity plots of bovine GPx1, inhibited by various pentathiepins and MSA at 23 °C (mean±SD); B) Residual relative GPx activity determined directly at the noted inhibitor concentrations or (a–f) after a 100‐fold jump‐dilution of incubations at the higher inhibitor concentration (i. e., 2.5 and 6.0 μM), pre‐incubated for 30 min at 23 °C under the following conditions: a) GPx (5 U/L)+inhibitor+250 μM GSH+500 μM *t*‐BHO; b) GPx (5 U/L)+inhibitor+250 μM GSH; c) GPx (5 U/L)+inhibitor; d) inhibitor+250 μM GSH+500 μM *t*‐BHO; e) Inhibitor+250 μM GSH; f) inhibitor alone. The dotted line represents the enzyme activity expected after the jump‐dilution if inhibition were to be 100 % reversible. *) *p*<0.01, two‐sided, paired *t*‐test, *n*=5.

The ability of pentathiepins to inhibit the bovine erythrocyte GPx activity was assayed for by an established enzyme method, which is based on the GPx‐dependent reduction of *tert‐*butyl hydroperoxide (TBHP) by GSH, leading to the formation of GSSG, which is coupled to the reduction of GSSG back to GSH by glutathione reductase (GR) and the oxidation of NADPH to NADP^+[10]^ (Figure 1). Inhibition potencies of the compounds were ranked by their IC_50_ values; that is, concentration of the inhibitor at the inflection point in the sigmoidal log dose‐inhibition curve (Figure 4A), and are summarized for the pentathiepins and mercaptosuccinic acid (MSA) in Table 1. All of the tested pentathiepins, regardless of the heterocyclic scaffold, showed a strong level of GPx inhibition, ranging from 3.76 down to 0.40 μM. All have greater potency than the best‐known inhibitor MSA, which has an IC_50_ of 5.86 μM in the same assay. In fact, one of the pentathiepins (**7**) was 15 times more potent at inhibiting GPx1 compared to MSA.

**Table 1:** Average IC_50_ values [confidence interval (CI) 95 %; *n*>3] at 23 °C for the inhibition of bovine erythrocyte GPx activity by mercaptosuccinic acid (MSA) and pentathiepins **1**–**8**, and average GI_50_ values (CI 95 %; *n*>4) at 37 °C for growth inhibition of the SISO human cervix cancer cell line.

Cmpd	Enzyme inhibition IC_50_/	Inhibition of cell viability GI_50_/
	[μM] (CI 95 %)	[μM] (Cl 95 %)
MSA	5.86 (4.21–8.14)	57.1 (47.6–68.3)
**1**	1.76 (1.39–2.24)	n.d.
**2**	0.52 (0.45–0.61)	n.d.
**3**	0.47 (0.38–0.59)	n.d.
**4**	2.44 (2.17–2.75)	8.9 (5.9–13.3)
**5**	1.86 (1.67–2.04)	n.d.
**6**	0.95 (0.90–0.99)	3.5 (2.6–4.7)
**7**	0.40 (0.23–0.69)	1.5 (1.3–1.8)
**8**	0.83 (0.62–1.13)	1.4 (1.3–1.5)

n.d.: not determined due to poor aqueous solubility

Several interesting selectivities for the compounds are noted. For the pyrrolo[1,2‐a]quinoxaline derivatives a methyl group para to the nitrogen of the pyrrol (**2**) increases potency compared to the unsubstituted analogue **1**. Adding the meta‐methyl group to **2** yielding the bis‐methyl derivative **4** results in a dramatic loss in potency. A trifluoromethyl‐ substituent at position R^3^ has no noticeable effect on inhibitory potency, either for the mono‐ or di‐methylated derivates (**3** or **5**) compared to **2** and **4**. In the cases of the indole derivatives **6**–**8**, which were all notably more potent than MSA (Figure 4A), neither substituents on the indole nitrogen nor the aromatic ring had a noticeable effect on potency. Thus, while pentathiepins with the indole scaffold are somewhat more potent at inhibiting GPx1 they lack the selectivity observed with the pyrrolo[1,2‐a]quinoxaline based pentathiepins.

To assess whether the inhibition of bovine GPx1 by **4** is reversible or irreversible/tight‐binding, we performed jump‐dilution experiments.^21^ For these studies, enzyme and inhibitor were first incubated at 100‐ and tenfold the IC_50_ concentrations, respectively, for 30 min at 23 °C, then rapidly diluted 100 times in assay buffer followed by the measurement of residual enzyme activity (Figure 4B). No difference in the inhibitory activity of **4** after jump‐dilution (Figure 4B left, a) was observed compared to expected inhibition at 0.25 μM (Figure 4B left, 0.25 μM), evidence that GPx1 inhibition is reversible. On the other hand, the inhibitory activity of MSA (Figure 4B right, a) was significantly greater than the level expected for a concentration of 0.60 μM MSA (Figure 4B right, 0.6 μM), evidence that some irreversible inhibition had occurred. This is consistent with findings that MSA can act as an irreversible inhibitor of GPx1.^11^ In further jump‐dilution experiments, we pre‐incubated both inhibitors with the assay concentrations of either GSH and TBHP (a, d) or GSH only (b, e), with (a–c) and without (d–f) GPx1 for 30 min. Again, no changes in the inhibitory activities after jump‐dilutions of **4** were apparent compared to the normal assay (Figure 4B left, d–f), evidence that assay concentrations of GSH and t‐BHP do not affect the inhibitory activity of **4**. Moreover, pre‐incubations of MSA without enzyme (Figure 4B right, d) did not result in significantly more inhibition of GPx during the assay compared to inhibition at 0.6 μM (Figure 4B right, 0.6 μM), evidence that irreversible inhibition by MSA is mechanism‐based.

To investigate whether the pentathiepins have an inhibitory effect on human GPx, cell lysates of the human cervix cancer cell line SISO, which expresses high levels of GPx1 (see below), were incubated with either **4** or MSA in concentrations of the IC_50_ value for the inhibitory effect on bovine GPx1. At the IC_50_ values previously obtained with bovine erythrocyte GPx (Table 1), compound **4** led to about 20 % statistically significant (*p*<0.001) inhibition of GPx activity while MSA was considerably more potent, giving a 60 % decrease in activity (*p*<0.001).

#### Pentathiepins do not inhibit other antioxidative enzymes

The possibility that the pentathiepins could inhibit other antioxidative enzymes was investigated by measuring the degree of inhibition of the following enzymes: rat thioredoxin reductase (TrxR), yeast glutathione reductase (GR), bovine catalase and bovine superoxide dismutase (SOD). None of these enzymes was inhibited to any appreciable extent by any of the compounds tested at concentrations of either 20 or 25 μM (Figures S33–S36); these concentrations are more than 20 times higher than the highest IC_50_ value for bovine erythrocyte GPx (Table 1). It was also of particular importance to rule out inhibition of yeast GR because this enzyme is an auxiliary enzyme in the GPx assay. Thus, these data provide evidence that the pro‐oxidative properties of pentathiepins observed with cells (see below) do not originate from their capability to inhibit these enzymes.

#### GPx activity and protein expression in different cancer cell lines

**Figure 5:**
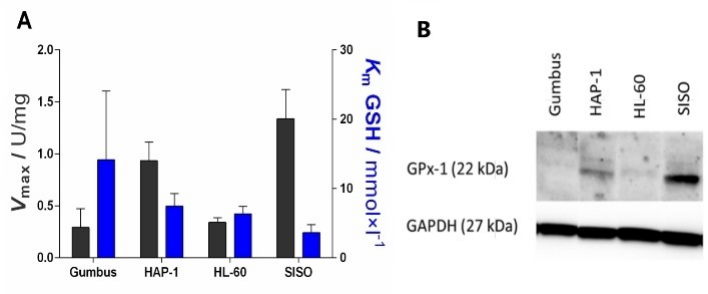
A) GPx activity of cancer cell lysates expressed as maximum velocity per mg [U/mg] (black) and corresponding Michaelis‐Menten constant (*K*_m_; blue; mean±SD, *n*>3) at 23 °C. B) Western blot analyses of human GPx1 in various cancer cell lines.

To better understand the relationship between GPx activity in cancer cells and the anti‐proliferative effects of pentathiepins, both the enzyme activity and protein expression of human GPx1 in four human cancer cells lines were determined. It was found that SISO cells have the highest enzyme activity followed by the human leukemia HAP‐1 cells, while the human B‐cell lymphoma line Gumbus and human leukemia line HL‐60 have similar low levels of activity (Figure 5A). The *K*_m_ values for GSH for all four cell lines were between 3.6–7.5 mm, which is in the range of the cellular concentrations of GSH in cancer cells.^22^ The data for the *V*_max_ of GPx correlates roughly to the protein expression profiles of these cells for GPx1, determined by western blotting (Figure 5B). The highest protein levels were found in the SISO cells followed by the HAP‐1 and HL‐60 cells. For Gumbus cells it was not possible to detect any expression of the GPx1 protein. This might be because the protein expression is below the level of detection by western blotting or due to the peroxidase activity stemming from other GPx enzymes, such as GPx‐4 in this cell line.

#### Pentathiepins inhibit the growth of human cancer cells

The exposure of the SISO cell line to various pentathiepins resulted in a dose dependent inhibition of the cell growth, as measured by the crystal violet assay. GI_50_ values for 50 % growth inhibition were all in a low micro molar range (Table 1). It was not possible to measure GI_50_ values for **1**–**3** and **5** because they are insoluble in the culture medium at concentrations above the estimated GI_50_ values. The indole−pentathiepins gave lower GI_50_ values than the pyrrolo[1,2‐a]quinoxaline derivatives. All of the indole derivatives lacking a substituent in position R^2^ exhibit quite similar GI_50_ values between 2.4–3.8 μM. There was also a trend showing that an electron rich substituent in position R^2^ increased the antiproliferative effect in SISO cells. In comparison to the pentathiepins, MSA had a much weaker anti proliferation effect with a GI_50_ value of 57.1 μM.

**Figure 6:**
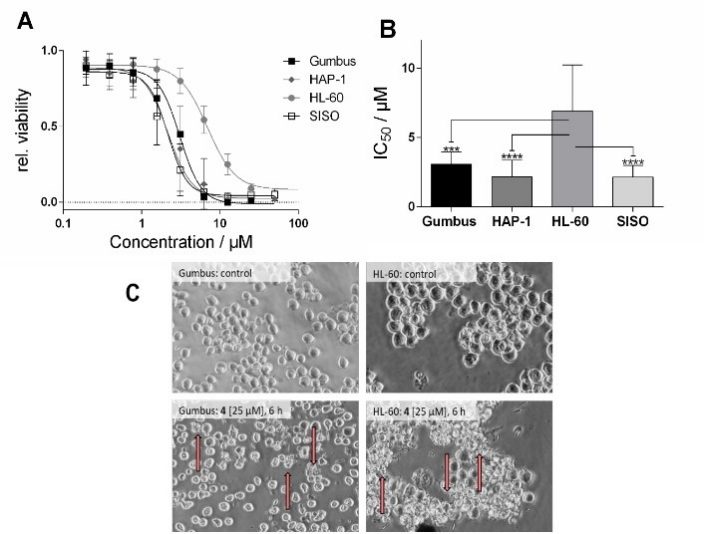
A) Relative cell viability after exposure to various concentrations of **4** for 48 h (mean±SD, *n*>3); B) IC_50_ values for the reduction of cell viability of **4** in various cell lines (mean±confidence interval 95 %, *n*=3; ***p<0.001; ****p<0.0001; C) Morphology of Gumbus and HL‐60 cells after exposure to 25 μM **4** for 6 h (induced membrane blebbing highlighted by arrows).

The exposure of various cell lines to **4** for 48 h resulted in a dose dependent decrease in cell viability, as measured by the MTT assay (Figure 6A). We determined IC_50_ values for the viability reduction in a low micro molar range (Figure 6B). While the Gumbus, SISO or HAP‐1 cell lines appear to have similar sensitivities to **4** with IC_50_ values around 2.2–3.1 μM, the HL‐60 lines is significantly less sensitive with an IC_50_ of 6.9 μM. Moreover, we found that at the highest tested concentration of **4**, complete loss of cell viability in Gumbus, SISO and HAP‐1 cells was achieved (Figure 6A).

#### Morphology of Gumbus and HL‐60 cells after incubation with 4

A dramatic change in morphology was observed for Gumbus and HL‐60 cells incubated with **4** at a concentration of 25 μM after only 6 h (Figure 6C). The treated cells have a smaller size compared to untreated cells while displaying membrane blebbing (marked with an arrow in Figure 6C), a hallmark of apoptosis.

#### Pentathiepin 4 causes a burst of reactive oxygen species in cancer cells

**Figure 7:**
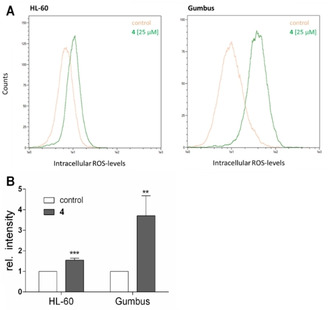
A) Representative flow cytometric histograms of ROS determination with DCF‐DA‐labeled HL‐60 (left) and Gumbus (right) cells after incubation with 25 μM **4** for 10 min (green) or vehicle control (orange) at *λ*_em/ex_=488/530 nm, B) Relative increase of ROS after incubation with 25 μM **4** for 10 min (mean+SD, *n*>4, ***p<0.001, ****p<0.0001).

It has long been observed that pentathiepins are able to produce reactive oxygen species outside of cells.^16^, ^17^, ^20^ Whether ROS production can also occur in living cells has not yet been reported. To determine if **4** can produce ROS in HL‐60 and Gumbus cells, the cytosolic ROS detector 2′,7′‐dichlorofluorescin diacetate (DCF−DA) was used. It was found that **4** induces a significant burst of ROS after just 10 min incubation with the Gumbus cell line (Figure 7). In the HL‐60 cell line, the burst of ROS was also rapid but not as intense as with the Gumbus line. Thus, pentathiepins appear capable of generating ROS inside living cells within a very short period of time.

#### Pentathiepin 4 disrupts mitochondrial membrane potential

**Figure 8:**
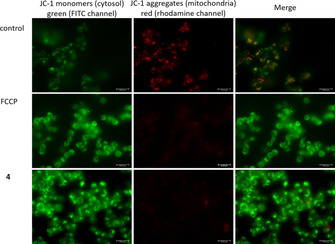
Fluorescence microscopy of HAP‐1 cells after incubation with FCCP or 2.2 μM **4** for 1 h followed by staining with JC‐1 dye; Left: JC‐1 monomers in cytosol, emission of green light (FITC channel); middle: JC 1 aggregates in mitochondria, emission of red light (rhodamine channel); right: overlay.

A burst of ROS could be an indication that mitochondria are adversely affected. To determine if **4** influences the mitochondrial membrane potential (MMP), the fluorescent dye JC‐1 was used together with fluorescence microscopy. Control cells showed both green and red fluorescence (Figure 8), indicating that JC‐1 is present in both the cytosol as well as the mitochondria. Following a 1 h incubation with positive control FCCP (final concentration: 2.5 μg/L) no red fluorescence was detectable, indicating complete loss of MMP. The exposure of the cells to **4** at concentrations of the IC_50_ for viability reduction for 1 h led to a similar loss of MMP. Thus, pentathiepins very rapidly disrupt MMP, which likely triggers cells to undergo apoptosis. Whether this event is related to the GPx inhibitory effect of **4** will require further study.

#### Pentathiepin 4 induces strand breaks in super‐coiled plasmid DNA

**Figure 9:**
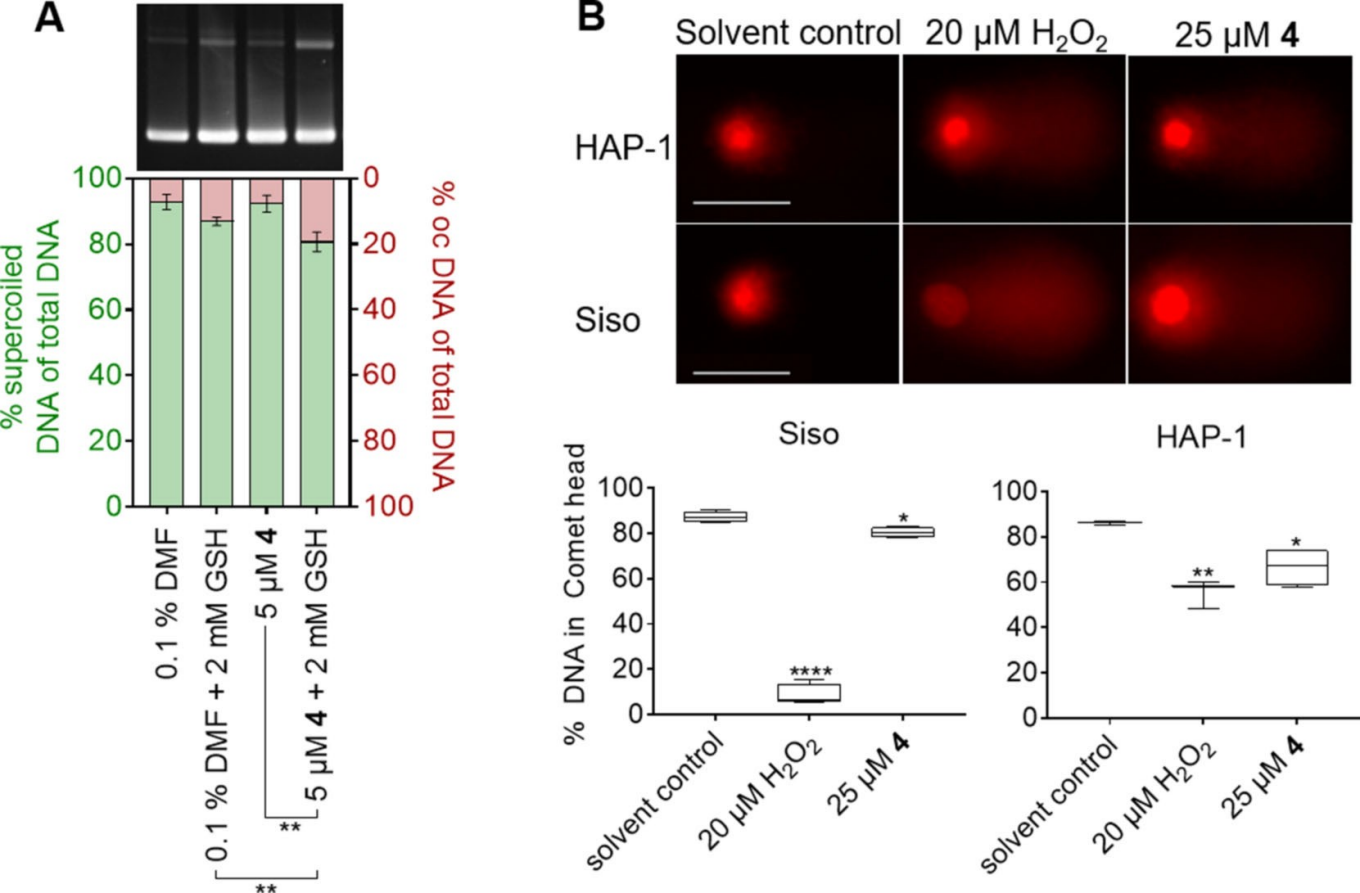
A) Supercoiled plasmid cleavage by compound **4** or the respective solvent DMF in the absence and presence of GSH, displayed as ratios of supercoiled (left *y*‐axis) and open circular (right *y*‐axis) plasmid DNA. Data from three independent replicates were compared by two‐tailed unpaired *t*‐test (*n*=3; **p<0.01). B) Above: Representative images of comets in HAP‐1 and SISO cells exposed to either 1 % DMF as solvent, H_2_O_2_ or **4** for 15 min at 0 °C. Scale bar: 50 μm. Below: Box and‐whiskers plot displaying the percentage of intact genomic DNA in the comet head after various treatments. One‐way ANOVA and Dunnett's multiple comparisons tests were performed relative to the solvent control (*n*≥3; *p<0.05; **p<0.01; ****p<0.0001).

To assess the potential DNA‐cleaving activity of **4** a plasmid cleavage assay was performed. This method makes use of the different mobilities that plasmids display during electrophoretic separation processes in an agarose gel. An intact supercoiled plasmid has the highest mobility due to its compactness, open circular plasmids (ocDNA), e. g. plasmids with a single strand break, present a lower mobility due to increased friction of this less compact DNA construct. Commercially available DNA plasmids usually present ratios of 95 % supercoiled and 5 % ocDNA. Upon treatment with DNA‐damaging agents this ratio will be shifted towards a higher amount of ocDNA due to single strand breaks, or even linearized DNA in case double strand breaks are induced. In both negative control samples with 0.1 % DMF with or without GSH the percentage of damaged DNA was 7 and 13 %, respectively (Figure 9A). When the plasmid was treated with 5 μM compound **4**, the fraction of damaged plasmid increased to 19 % in presence of 2 mm GSH but remained at 7 % without additional thiol in the reaction. Thus, **4** together with intracellular concentrations of GSH leads to a significant increase in DNA cleavage.

#### Pentathiepin 4 induces DNA strand breaks in cancer cells

To assess the induction of DNA strand breaks by pentathiepin **4** the Comet Assay was used. The SISO and HAP‐1 cell lines were treated with either 1 % DMF (solvent control), 20 μM H_2_O_2_ (positive control) or 25 μM **4** for 15 min at 0 °C. The DNA in the comet head corresponds to still intact genomic DNA while the tail forms due to the damage of nuclear DNA (Figure 9B). For both cell lines H_2_O_2_ resulted in significant decreases of intact DNA, whereby SISO cells were more affected than the HAP‐1 cells (Figure 9B). Exposure of SISO cells to **5** resulted in a small but significant decrease in the percentage of undamaged DNA from 87 % in control cells to 80 % in treated cells. For HAP‐1 cells a greater reduction of intact DNA from 86 % in control cells to 66 % in pentathiepin‐incubated cells was observed. Interestingly, the effects of H_2_O_2_ and **4** on comet tail formation opposed each other in the two cell lines; i. e., H_2_O_2_ caused larger comets in SISO cells whereas **4** caused larger comets in HAP‐1 cells.

#### Pentathiepin 4 induces apoptosis in cancer cells

**Figure 10:**
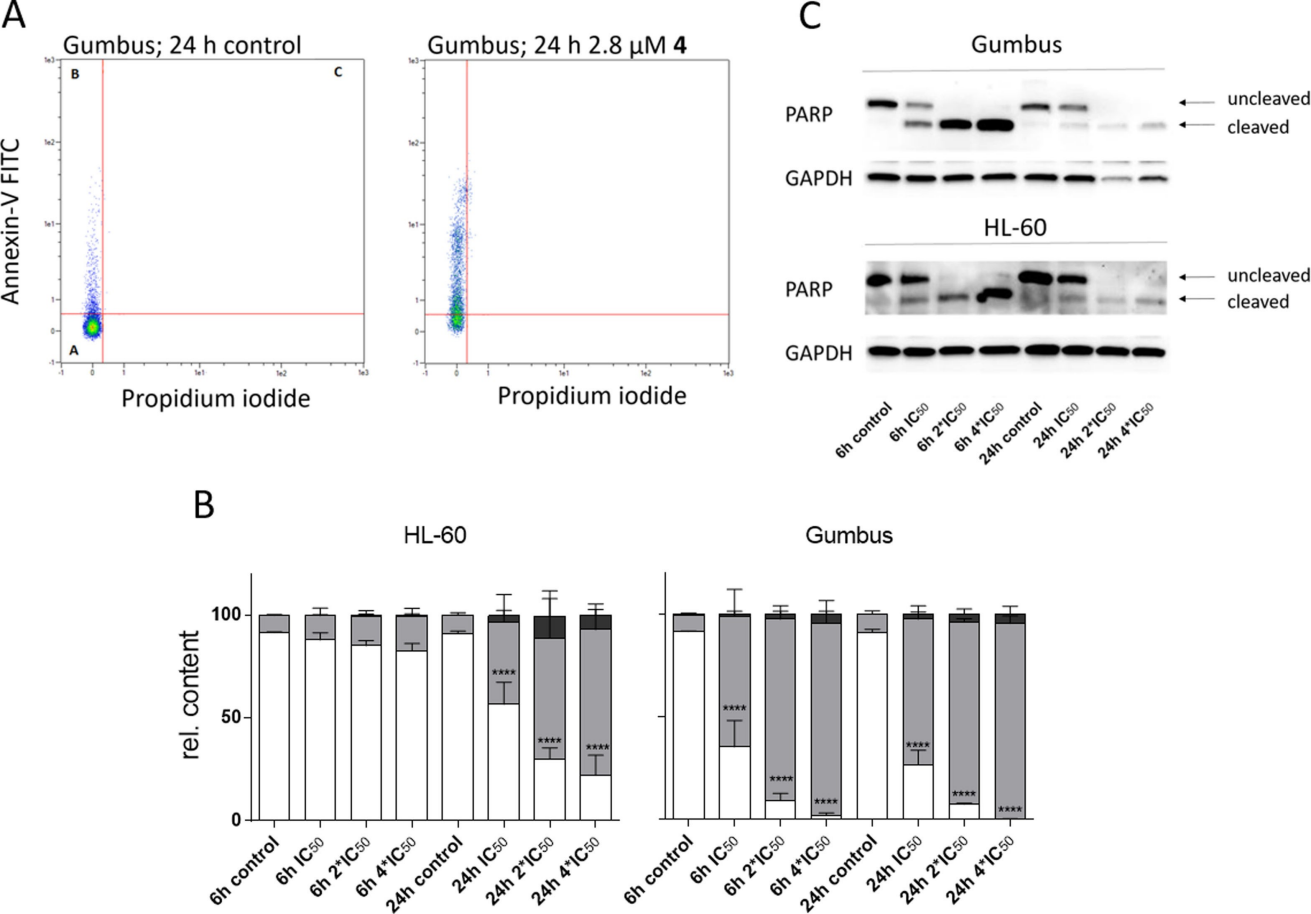
A) Representative flow cytometric plots of Annexin‐V/PI‐labeled Gumbus cells, with and without exposure to 2.8 μM **4** for 24 h (population in quadrant I: living cells; II: apoptotic cells; III: late apoptotic/necrotic cells), B) Results of cytometric studies with Annexin‐V/PI‐labeled HL‐60 and Gumbus cells treated with **4** at multiple concentrations of the viability IC_50_ for 6 or 24 h; living cells (white), early apoptotic cells (gray), late apoptotic/necrotic cells (black; mean+SD, *n*=3, ****p<0.0001); C) Detection of PARP cleavage by western blotting in Gumbus and HL‐60 cells after incubation with various concentrations of **4** for 6 or 24 h.

To determine whether the loss in viability by **4** is caused by apoptosis, a flow cytometric analysis with Annexin‐V−FITC and PI‐stained cells was used. Early apoptotic cells undergo a translocation of phosphatidyl serine from the inner to the outer cell membrane monitored by Annexin‐V−FITC. Late apoptotic or necrotic cells also show a loss of cell membrane integrity verifiable by PI. Cells were exposed to **4** in multiple concentrations of the viability inhibition IC_50_ for 6 and 24 h. Already after 6 h incubation time a significant increase of an apoptotic population in the Gumbus cell line was detected (Figure 10A), which is consistent with the morphology studies. With the fourfold IC_50_ concentration nearly every cell was apoptotic after 6 h. The HL‐60 cells showed a slower induction of apoptosis but after 24 h a significant increase of an apoptotic population was also detected (Figure 10B).

Another method for detecting caspase dependent apoptosis is to measure the cleavage of the enzyme poly‐(ADP‐ribose)‐polymerase (PARP) by western blotting. After an apoptotic stimulus, caspases, e. g. caspase‐3 and caspase‐7, cleave PARP, resulting in a loss of its function to mediate DNA‐repair processes. Cells were incubated with multiple IC_50_ concentrations of **4** for 6 and 24 h. Figure 10C shows that after just 6 h exposure time cleavage of PARP was detectable via western blotting in both Gumbus and HL‐60 cells in a concentration dependent manner. Thus, pentathiepin **4** appears to induce the caspase dependent induction of apoptosis.

#### Pentathiepin 4 does not induce ferroptosis

Another form of programmed cell death is ferroptosis, which is iron dependent and activated by lipid peroxidation (LPO) in the cell membrane.^14^ Unlike apoptosis, PARP cleavage is not observed in this cell death mechanism. GPx4, a membrane bound isoenzyme of GPx1, is believed to be a key protector of cells from ferroptosis by destroying lipid peroxides in the cell membrane. Recently, inhibitors of GPx4 have been found that initiate ferroptosis and may also find use as anticancer drugs.^15^, ^23^, ^24^ If pentathiepins were to inhibit GPx4 in the cell membranes, some of the cytotoxic effects of these compounds could be the result of ferroptosis as well as apoptosis. To probe for the possible inhibition of GPx4 by pentathiepins and with that the subsequent induction of ferroptosis, we treated cells with cytotoxic concentrations of **4** together with a known ferroptosis inhibitor, ferrostatin‐1.^25^ No significant changes in the IC_50_ value of **4** were observed when co‐treated with ferrostatin‐1 at concentrations between 1.5 and 6.0 μM in the SISO cell line (Figure S37), evidence that **4** does not induce ferroptosis.

In another set of experiments, LPO was visualized by fluorescence microscopy with a dye for lipid peroxides, BODIPY 581/591. Although the positive control, *tert*‐butylperoxide (*t*BuOOH), a known inducer of ferroptosis,^26^ showed LPO in SISO cells after 24 h, we found no indication for LPO in cells treated with cytotoxic concentrations of **4** after 24 h (Figure S38), thus ruling out an involvement of LPO in the death of SISO cells. Together, our results point to apoptosis and not ferroptosis as the mechanism of cell death induced by **4**.

## Discussion

Glutathione peroxidase represents a family of key antioxidative enzymes acting to protect both normal and cancerous cells from the toxic effects of oxidative stress. Rapidly dividing cells, such as cancer cells, are more vulnerable to oxidative stress due to elevated levels of metabolism. By interfering with antioxidative systems such as inhibition of GPx, one might expect stronger cytotoxic activity towards cancer cells than normal cells. This hypothesis has guided us to discover a new class of GPx inhibitors, the pentathiepins, which are able to inhibit bovine erythrocyte GPx activity at concentrations more than 10 times lower than the until now most potent known inhibitor, MSA.^10^ In fact, the most potent inhibitor discovered here (**7**) is ca. 15 times more potent than MSA. Even the least potent pentathiepin (**4**) was still twice as potent as MSA (IC_50_ 2.44 μM compared to 5.86 μM) against the bovine enzyme.

Some structure‐effect relationships with regard to GPx inhibition were apparent. For the pyrrolo[1,2‐a]quinoxaline derivatives, a methyl substituent in para position to the pyrrolo nitrogen and an electron rich substituent in meta lead to noticeable increases in potency. In the case of the indole derivatives, which were generally more potent than the pyrrolo[1,2‐a]quinoxaline derivatives, no selectivity war noted. To assess whether pentathiepins are also capable of inhibiting GPx from human sources, the ability to inhibit GPx activity from human cancer cell lysates of SISO cells was investigated. The SISO cell line, which expresses the highest levels of GPx amongst all cell lines employed in this study (see above Figure 7A and B), was used as a source of GPx. The traditional inhibitor MSA effectively inhibited human GPx at approximately the same potency as the bovine enzyme while the representative pentathiepin **4** was a weaker inhibitor of the human compared to the bovine GPx. This could be a result of weaker inhibition of the human enzyme compared to the bovine enzyme. On the other hand, it could also be due to the much greater lipophilicity of **4** compared to MSA, causing a considerable fraction of the pentathiepin to bind to cellular proteins other than GPx, thus reducing the free fraction available for enzyme inhibition.

It has been reported previously that various pentathiepins possess anticancer activity in vitro.^14^, 15b Similarly, all of the new pentathiepins tested here possess cytotoxic activity on various human cancer cell lines, with IC_50_ values ranging from low micro molar to sub‐micro molar concentrations. In fact, some of the pentathiepins show comparable antiproliferative potency to anticancer drugs used in cancer therapy.^8^, ^22^

We focused our biological investigations on the activity of a pentathiepin from the pyrrolo[1,2‐a]quinoxaline class (i. e., **4**) because these derivatives showed more distinct selectivity with regards to GPx1 inhibition compared to the indole based pentathiepins, and thus they could be acting more specifically in cells. In the case of **4**, there appears to be a reciprocal relationship between the levels of GPx1 expressed in the four cell lines (as well as the levels of cellular enzyme activity) and cytotoxic potency; that is, cell lines SISO and HAP‐1 which express higher levels of GPx1 (or higher GPx activity) were more sensitive to the cytotoxic effects of **4** than HL‐60 and Gumbus cells, which both expresses lower levels of the enzyme (compare Figure 7A/B with Figure 8). This suggests that HL‐60 and Gumbus cells might rely on other antioxidative mechanisms than GPx while SISO and HAP‐1 are strongly dependent on GPx for antioxidative protection.

Based on studies with isolated DNA, the mode of action for pentathiepins has been postulated to involve the formation of hydrogen peroxide, which is further activated in the Fenton reaction to form hydroxyl radicals, provoking DNA oxidation and strand breaks.^16^, ^17^ We were able to confirm that compound **4** likewise causes DNA strand breaks in super‐coiled plasmid DNA. However, data on the *in vivo* mechanism of pentathiepin cytotoxicity has been lacking until now. Here we report that **4** can indeed bring about a burst of ROS in cells, whereby the Gumbus cells display much higher ROS levels than HL‐60 cells. The enhanced levels of ROS could be explained on the one hand by the inhibition of GPx1, which could lead to a ROS accumulation, while on the other hand polysulfides, resulting from a reaction with thiols such as GSH, are known to bring about the formation of H_2_O_2_ and subsequently hydroxyl radicals that can cleave nuclear DNA.^27^ Indeed, we observed with the Comet assay significant increases in strand breaks of DNA only 15 min after exposure of cancer cells to **4**.

To investigate the specificity of inhibition of GPx1, the abilities of various pentathiepins to inhibit other antioxidative enzymes such as SOD, catalase, TrxR and GR were investigated. It was important to establish that the antioxidative activity of the pentathiepins is not a result of inhibition of either SOD or catalase, which decomposes superoxide and hydrogen peroxide, respectively, in cells. It is known that some inhibitors of GPx1 such as gold and mercury compounds also inhibit GR and/or TrxR.12b, 28 TrxR also utilize an active‐site selenocysteine so it was imperative to rule out inhibition of this important enzyme class. It was found that none of these enzymes was inhibited by any of the tested compounds at concentrations up to 25 μM.

GSH is the predominant thiol in living cells and has a concentration around 4 mm in both HL‐60 and Gumbus cells.^29^ An explanation of the lower levels of ROS in HL‐60 cells could be due to the much higher activity of catalase reported in HL‐60 cell lines.^29^ Lee et al. have shown that catalase can prevent DNA‐strand breaks by pentathiepins on isolated supercoiled plasmid DNA *in vitro*.16a We have previously found that HL‐60 cells have four time higher catalase activity than Gumbus cells,^29^ which can lead to a swift degradation of hydroperoxides formed by pentathiepins. This would also explain why the HL‐60 cells are the least sensitive towards **4**.

**Figure 11:**
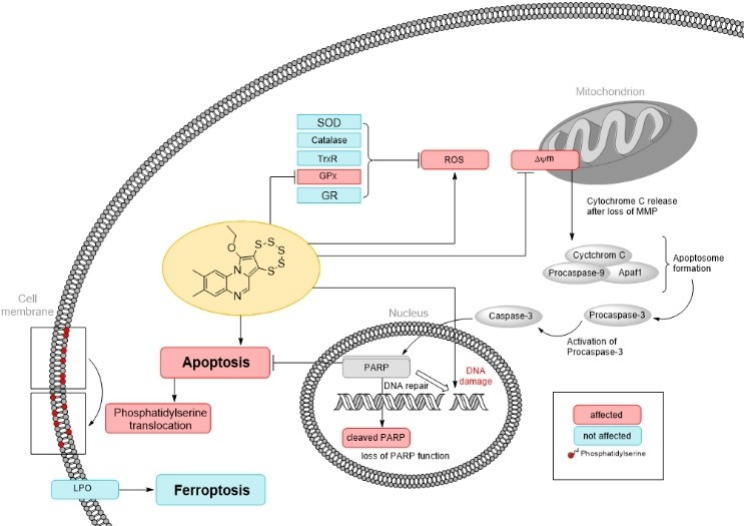
Summary of the proposed biological effects of pentathiepin **4** in cancer cells. Red:affect, blue: not affected

Figure 11 summarizes our findings on the cellular effects of pentathiepin **4**. Whether the inhibition of cellular GPx1 is directly involved in these events is not yet known with certainty. However, oxidative stress and loss of mitochondrial membrane potential appear to be central to the cytotoxic activity of compound **4**. It is well known that oxidative stress induces apoptosis.^30^ This might be the cause of strand breaks of nuclear DNA, which was detected in the Comet assay with **4**. For this compound, apoptosis in the Gumbus and HL‐60 lines was detected by the Annexin‐V and PARP cleavage assays as well as by morphological changes in treated cells. The induction of apoptosis in both cell lines was rapid, as evidenced by changes in cell morphology and PARP cleavage within just 6 h of exposure to **4**. The activation of PARP cleavage is consistent with apoptosis via the intrinsic pathway. However, when measuring apoptosis by the Annexin‐V assay, HL‐60 cells showed a delayed onset of apoptosis in comparison to Gumbus cells. This indicates that cancer cell lines react differently to the cytotoxic effects of pentathiepins. Apoptosis would appear to be initiated by the rapid loss in mitochondrial membrane potential (MMP), detected shortly after exposure of HAP‐1 cells to **4**. On the other hand, evidence for lipid peroxidation and cell death by ferroptosis was lacking, indicating that **4** does not act by inhibiting membrane bound GPx4.

## Conclusion

We have identified pentathiepins as a new class of potent inhibitors of GPx1. These compounds cause oxidative stress in cancer cells, resulting in DNA strand breaks, induction of apoptosis and cell death. To the best of our knowledge, this is the first time that the induction of oxidative stress, DNA strand breaks and apoptosis in cancer cells has been reported for pentathiepins. On the other hand, LPO is not involved in cell death, ruling out ferroptosis as a form of cell death. Interestingly, cell lines with higher levels of GPx1 expression appear to be more sensitive to pentathiepins than cells with lower levels. The loss of MMP appears to play a key role in the mechanism of action of pentathiepins. Ongoing studies are aiming to establish a link between GPx1 inhibition and cytotoxicity.

## Experimental Section

The completely dried *N,N‐*dimethylformamide (DMF, 99.8 %, extra dry, stored over molecular sieves) was purchased from Acros organics and used as received for all air or moisture sensitive reactions. Chloroform was freshly distilled before use. Machery‐Nagel silica gel 60 F254 plates were used for thin layer chromatography (TLC) and detection was achieved by UV light. Column chromatography was performed on Machery‐Nagel silica gel 60 (40–63 μm) or on Acros Organics silica gel 60 (35–70 μm). Unless otherwise noted, all biochemicals were from Sigma‐Aldrich (Taufkirchen, Germany). RPMI1640 culture medium was obtained from PAN‐Biotech (Aldenbach, Germany). Fetal bovine serum (FCS), penicillin/streptomycin, 3‐(4,5‐dimethyl‐2‐thiazolyl)‐2,5‐diphenyl‐2*H*‐tetrazolium‐bromide (MTT) 2,7‐dichlorofluorescein diacetate (DCF−DA), BODIPY 581/591, ferrostatin‐1, glutathione, glutathione disulfide, glutathione reductase, glutathione peroxidase, catalase, superoxide dismutase, mercapto succinic acid and the secondary antibody anti‐rabbit‐HRP were purchased from Sigma Aldrich (Taufkirchen, Germany). DMSO (for cell culture) and NAPDH were purchased from Carl‐Roth (Karlsruhe, Germany). The Annexin‐V apoptosis kit was obtained from Miltenyi Biotec (Teterow, Germany) and the mitochondrial apoptosis staining kit from Promokine (Heidelberg, Germany). The primary antibodies anti‐GPx1, anti‐PARP and anti‐GAPDH were purchased from Cell Signalling Technology (Cambridge, UK).

Melting points were determined with a Büchi Melting Point M‐565 or a Büchi 545 (Büchi, Flawil, CH), temperatures are uncorrected. ^1^H NMR and ^13^C NMR spectra were recorded either with a Bruker Avance II‐300 or an Avance II Ultrashield 400 MHz instrument. Chemical shifts *δ* are given in ppm and the solvent residual peak (CDCl_3_: ^1^H, *δ*=7.27; ^13^C, *δ*=77.0 and [D_6_]DMSO: ^1^H, *δ*=2.50; ^13^C, *δ*=40) was used as an internal standard with the quinoxaline derivatives while TMS was used for the indole derivatives. APCI‐MS (m/z) spectra were recorded on an Advion MS. HPLC were performed with a Merck‐Hitachi LaChrom 7000 instrument fitted with a Merck Chromolith SpeedROD RP‐18e column (4.6×50 mm). Samples of 25 μL were injected and eluted with a solvent of 80 % acetonitrile/water at a flow rate of 1.0 and 1.25 mL/min for purity and stability testing, respectively. Detection was done between the wavelengths of 210 and 500 nm; purities were estimated at *λ*=250 nm; all compounds showed ≥95 % purity by this method. Elemental analyses (C, H, N and S) were carried out by using an Elementar Vario Micro Cube elemental analyzer.

Single crystal X‐ray structural data were collected at RT (**5**) or low temperature (−103.0 °C; **8**) using a STOE‐IPDS 2T diffractometer with graphite‐monochromated molybdenum Kα radiation, *λ*=0.71073 Å. The structures were solved by direct methods (SHELXS‐13 and SIR‐2014) and refined by full‐matrix least‐squares techniques (SHELXL‐13).^31^ All non‐hydrogen‐atoms were refined with anisotropic displacement parameters. The hydrogen atoms were refined isotropically on calculated positions using a riding model with their U*iso* values constrained to 1.5 Ueq of their pivot atoms for terminal sp^3^ carbon atoms and 1.2 times for all other carbon atoms. The crystals of **5** were nonmerohedral two‐domain twins and refined with a hklf5 file. The data quality was poor, but the molecular structure of compound **5** was unambiguously established. CCDC1858786 (**5**) and 1858785 (**9**) contain the supplementary crystallographic data for this paper. These data are provided free of charge by The Cambridge Crystallographic Data Centre.

**Synthesis of pentathiepino[6′,7′ : 3,4]pyrrolo[1,2‐a]quinoxaline derivatives**: Compounds **1** and **4** were synthesized previously according to a reported protocol by Zubair et.al.;^32^ purities of 99.1 and 100 %, respectively, were confirmed by RP‐HPLC (Figures S1 and S2). The general synthetic procedure for **2**, **3** and **5** was: An oven dried 25 mL Schlenk flask was charged with the alkyne precursor, 0.5 equiv. of (Et_4_N)_2_[*M*oO(S_4_)_2_] and 1 equiv. of elemental sulfur under inert gas atmosphere (N_2_). The mixture was dissolved in a dry polar non‐protonating organic solvent (DMF or CH_3_CN) and allowed to react while stirred at 50 °C. The reaction progress was monitored by TLC. After the reaction was completed the crude product mixture was concentrated under reduced pressure and purified by silica gel column chromatography with ethyl acetate/hexane (5 to 20 %) as mobile phase.

**12‐Ethoxy‐3‐methyl‐[1,2,3,4,5]pentathiepino[6′,7′ : 3,4]pyrrolo[1,2‐a]quinoxaline (2)**: The general procedure was followed with the reagents 2‐(3,3‐diethoxyprop‐1‐ynyl)‐6‐methylquinoxaline (600 mg, 2.25 mmol), (Et_4_N)_2_[*M*oO(S_4_)_2_] (0.5 equiv, 709 mg, 1.13 mmol) and S_8_ (1 equiv, 578 mg, 2.25 mmol) in dry CH_3_CN. This reaction yielded 432 mg (1.125 mmol, 50 %) of a yellow solid. mp: 246–248 °C. ^1^H NMR (300 MHz, CDCl_3_) δ: 1.57–1.66 (m, 3 H, CH_3_), 2.51 (s, 3 H, C(6) CH_3_) 4.63–4.71 (m, 2 H, CH_2_), 7.34 (dd, *J*=8.50, 2.08 Hz, 1H, C(3)H), 7.74 (s, 1 H, C(5)H), 8.47 (d, *J*=8.31 Hz, 1 H, C(7)H), 8.86 (s, 1 H, C(8)H); ^13^C NMR (75 MHz, CDCl_3_) δ ppm : 15.92 (CH_3_), 21.44 (C(3) CH_3_), 73.62 (CH_2_), 116.72 (C6′,C6b), 117.09 (C7′,C11a), 122.66 (C1), 128.06 (C2), 129.78 (C4), 130.13 (C3), 130.36 (C7a), 136.94 (C7), 139.19 (C6), 143.93 (C4a), 144.83 (C12); (+ve) APCI‐MS *m/z* 384.58 [*M* ^+^] calcd. for C_14_H_12_N_2_OS_5_ [*M*]^+^, found: 385.44 [*M*+H]^+^. HPLC: *t*_R_=3.82 min, purity (@ *λ*=250 nm)=94.9 %. CHNS calc. C, 43.72; H, 3.15; N, 7.28; S, 41.69 found C, 43.83; H, 3.05; N, 7.08; S, 42.09.

**12‐Ethoxy‐3‐methyl‐6‐(trifluoromethyl)‐[1,2,3,4,5]pentathiepino[6′,7′ : 3,4]pyrrolo[1,2‐a]quinoxaline (3)**: The general procedure was followed by using 2‐(3,3‐diethoxyprop‐1‐ynyl)‐6‐methyl‐3‐(trifluoromethyl)‐quinoxaline (500 mg, 1.48 mmol), (Et_4_N)_2_[*M*oO(S_4_)_2_] (0.5 equiv, 465 mg, 0.739 mmol) and S_8_ (1 equiv, 417 mg, 1.48 mmol) in dry DMF yielding 421 mg (0.932 mmol, 63 %,) of a bright yellow solid. mp: 305–306 °C. ^1^H NMR (300 MHz, CDCl_3_) δ: 1.62 (m, *J*=5.96 Hz, 3H, CH_3_), 2.57 (s, 3 H, C(6)CH_3_), 4.60–4.68 (m, 2 H,CH_2_), 7.89 (d, *J*=8.25 Hz, 1 H,C(8)), 8.50–8.64 (m, 2 H,C(7),C(5)); ^13^C NMR (75 MHz, CDCl_3_) δ: 15.1 (CH_3_), 20.48 (C(3)CH_3_), 73.0 (CH_2_), 115.9 (C6a), 116.9 (C7′,C11a), 117.2 (C6′,C6b), 124.9 (q, *J*= 244.5 Hz, CF_3_), 126.6 (C1), 130.2 (C2), 130.3 (C4), 131.8 (C3), 133.7 (C7a), 136.7 (C4a), 141.1 (C6), 147.0 (C12); ^19^F NMR (282 MHz, CDCl_3_) δ: −63.3 (s, 1 F); (+ve) APCI‐MS *m/z* 452.58 [*M* ^+^] calcd. for C_15_H_11_F_3_N_2_OS_5_, found: 453.34 [*M*+H]^+^. HPLC: *t*_R_=4.76 min, purity (@ *λ*=250 nm)=100 %. CHNS calc. C, 39.81; H, 2.45; N, 6.19; S, 35.42 found C, 40.12; H, 2.62; N, 5.90; S, 35.98.

**12‐Ethoxy‐2,3‐dimethyl‐6‐(trifluoromethyl)‐[1,2,3,4,5]pentathiepino[6′,7′ : 3,4]pyrrolo[1,2‐a]quinoxaline (5)**: The general procedure was followed by using 2‐(3,3‐diethoxyprop‐1‐ynyl)‐6,7‐dimethyl‐3‐(trifluoromethyl)quinoxaline (500 mg, 1.42 mmol), (Et_4_N)_2_[*M*oO(S_4_)_2_] (0.5 equiv, 446 mg, 0.71 mmol) and S_8_ (1 equiv, 363 mg, 1.42 mmol) in dry CH_3_CN giving 364 mg (0.78 mmol, 55 %) of (micro‐) crystalline yellow needles. mp: 322–325 °C. ^1^H NMR (300 MHz, CDCl_3_) *δ* 1.63 (t, *J*=7.18 Hz, 4 H) 2.41 (s, 3 H) 2.48 (s, 3 H) 4.51–4.75 (m, 2 H) 7.79 (s, 1 H) 8.51 (s, 1 H); ^13^C NMR (75 MHz, CDCl_3_) δ: 15.4 (s, CH_3_), 19.5 (C(1)CH_3_), 20.9 (C(2)CH_3_), 73.5 (CH_2_), 110.7 (C6a), 117.0 (C6′,C7′: C6b, C11a,), 118.6 (C1,C4), 125.0 (q, CF_3_, J= 215.1), 131.0 (C2,C3), 132.5 (C7a), 136.3 (C4a), 140.6 (C6), 157.9 (C12); ^19^F NMR (282 MHz, CDCl_3_) δ ppm: −63.3 (s, 1 F); (+ve) APCI‐MS *m/z* 466.61 [*M* ^+^] calcd. for C_16_H_13_F_3_N_2_OS_5_ [*M*]^+^, found: 467.16 [*M*+H]^+^. HPLC: *t*_R_=6.45 min, purity (@ *λ*=250 nm)=100 %. CHNS calc. C, 41.18; H, 2.81; N, 6.00; S, 34.36 found C, 41.05; H, 2.66; N, 5.93; S, 34.69. X‐ray structural analysis: formula C_16_H_13_F_3_N_2_OS_5_, formula weight 466.58, crystal system: monoclinic; space group: *P*2_1_/*c*; unit cell parameters: *a*=18.941(4) Å, *b*=11.048(2) Å, *c*=9.2081(18) Å, *α*=90°, *β*=94.92(3)°, *γ*=90°; temperature of data collection: 293(2) K; *Z*, calculated density: 4, 1.614 g cm^−3^; *R* ^1^: 0.1880; GOF: 1.223.

**Synthesis of the indole derivatives**: The compounds **6**–**8** were synthesized as reported previously with modifications.^19^ General procedure: The indole was dissolved in 32 times the volume of freshly distilled CHCl_3_ and cooled to −22 °C by using an ice/ethanol mixture. To the stirred solution was added dropwise 0.80 molar equivalent of S_2_Cl_2_ and the reaction mixture was allowed to warm to 0 °C, then held at that temperature for 48 h. The reaction was refluxed for 3 h followed by filtration through a 4 cm column of kieselgur. The volume of the filtrate was concentrated under reduced pressure and the concentrate chromatographed on a silica gel column by using a mixture of CH_2_Cl_2_ in light petroleum as the eluent.

**6‐Methyl‐6H‐[1,2,3,4,5]pentathiepino[6,7‐b]indole (6)**: The general procedure was followed with the reagents *N*‐methyl indole (2.55 g, 20 mmol) and S_2_Cl_2_ (1.2 mL, 16 mmol) in 50 mL chloroform. The yellow filtrate was stored at 4 °C. After silica gel column chromatography (light petroleum/CH_2_Cl_2_ 8.5 : 1.5) 618 mg (2,13 mmol, 33.3 %) of a yellow solid was obtained. mp 122 °C (lit. mp 123–124 °C^19^). ^1^H NMR (400 MHz, CDCl_3_,) *δ*: 3.91 (s, 3H, CH_3_), 7.25–7.36 (m, 3H, C(9)H and C(8)H and C(7)H), 7.68–7.71 (2 × t, 1H, C(10)H). ^13^C NMR (100 MHz, CDCl_3_) *δ*=31.6 (CH_3_), 110.5 (C7), 119.3 (C10b), 120.7 (C10), 122.2 (C9), 124.7 (C8), 129.0 (C10a), 136.7 (C6a), 141.42 (C5a). (+ve) APCI‐MS *m/z* 289.92 [*M*+H^+^] calcd. for C_9_H_7_NS_5_ [*M*], found: 290.0 [*M*+H^+^], 226.1 [*M*+H^+^−2S], 194.0 [*M*+H^+^−3S], 162.1 [*M*+H^+^−4S]. HPLC: *t*_R_=2.85 min, purity (@ *λ*=250 nm)=95.3 %. CHNS calc. C 37.37, H 2.48, N 4.84, S 55.39; found C 37.33, H 2.61, N 4.88, S 56.96.

**9‐Chloro‐6‐ethyl‐6H‐[1,2,3,4,5]pentathiepino[6,7‐b]indole (7)**: The general procedure was followed with the reagents 5‐chloro‐*N*‐ethylindole (1.40 g, 8 mmol) and S_2_Cl_2_ (0.50 mL, 6 mmol) in 20.0 mL chloroform. The solvent was removed under reduced pressure and the crude product stored at 4 °C. After silica gel column chromatography (light petroleum/CH_2_Cl_2_ 9 : 1) 131 mg of a yellow solid was obtained (0.39 mmol, 15.6 %) mp 121.9 °C. ^1^H NMR (400 MHz, CDCl_3_): *δ*=1.39 (t, 3H, 3 J=7.2 Hz, CH3), 4.35 (m, 1H, J=7.2 Hz, CH_2_), 4.46 (m, 1H, J=7.2 Hz, CH_2_), 7.23–7.29 (m, 2H, C(8)H and C(7)H), 7.68 (m, 1H, C(10)H). ^13^C NMR (100 MHz, CDCl_3_) *δ*: 16.4 (CH_3_), 40.6 (CH_2_), 111.9 (C7), 118.8 (C10b), 120.4 (C10), 125.4 (C8), 128.2 (C9), 130.3 (C10a), 134.9 (C6a), 142.1 (C5a). (+ve) APCI‐MS *m/z* 337.89 [*M*+H^+^] calcd. for C_10_H_8_ClNS_5_ [*M*], found: 338.1 [*M*+H^+^], 274.0 [*M*+H^+^−2S], 242.1 [*M*+H^+^‐−3S], 210.1 [*M*+H^+^−4S]. HPLC: *t*_R_=5.31 min, purity (@ λ=250 nm)=96.9 %. CHNS calc. C 35.54, H 2.39, N 4.14, S 47.44; found C 35.47, H 2.54, N 4.15, S 47.83

**6‐Benzyl‐6H‐[1,2,3,4,5]pentathiepino[6,7‐b]indole (8)**: The general procedure was followed with the reagents *N*‐benzylindole (2.07 g, 10 mmol) and S_2_Cl_2_ (0.64 mL, 8 mmol) in 25.0 mL chloroform. The yellow filtrate was stored at 4 °C. After silica gel column chromatography (light petroleum/CH_2_Cl_2_ 9 : 1) the solvent evaporated at room temperature and atmospheric pressure. A yellow, crystalline solid was obtained (180 mg, 0.49 mmol, 15.3 %, mp 165.5 °C). ^1^H NMR (400 MHz, CDCl_3_): *δ*=5.57 (m, 2H, CH_2_), 7.05 and 7.26 (m, 8H), 7.70 (m, 1H, C(10)H). ^13^C NMR (100 MHz, CDCl_3_) *δ*: 48.8 (CH_2_), 111.3 (C7), 120.1 (C10b), 121.0 (C10), 122.6 (C9), 125.2 (C8), 126.7 (C Ar), 128.0 (C Ar), 128.7 (C Ar), 129.1 (C Ar), 129.5 (C10a), 136.5 (C6a), 141.9 (C5a). (+ve) APCI‐MS *m/z* 365.95 [*M*+H^+^] calcd. for C_15_H_11_NS_5_ [*M*], found: 366.1 [*M*+H^+^], 302.1 [*M*+H^+^−2S], 270.1 [*M*+H^+^−3S], 238.2 [*M*+H^+^−4S]. HPLC: *t*_R_=4.16 min, purity (@ *λ*=250 nm)=95.4 %. CHNS calc. C 49.28, H 3.03, N 3.83, S 43.86; found C 49.37, H 3.28, N 3.85, S 43.92. X‐ray structural analysis: formula: C_15_H_11_NS_5_, formula weight 365.55, crystal system: triclinic; space group: *P*‐1; unit cell parameters: *a*=8.7059(17) Å, *b*=9.768(2) Å, *c*=10.084(2) Å, *α*=108.20(3)°, β=102.15(3)°, *γ*=102.42(3)°; temperature of data collection: 170(2) K; *Z*, calculated density: 2, 1.599 g cm^−3^; *R*: 0.0389; GOF: 1.050.

**GPx enzyme activity assay**: To evaluate if the various compounds could inhibit GPx1, an enzymatic assay monitoring the activity of the bovine erythrocyte GPx was used as described previously.^33^ Bovine erythrocyte GPx is highly homologous to the human GPx1 (homology of 87 %)^34^ but much more affordable. In this assay, GPx reduces *tert*‐butylhydroperoxide (TBHP) followed by oxidation of glutathione (GSH) to the disulfide (GSSG), which in turn is regenerated to GSH by glutathione reductase (GR) with the consumption of NADPH. To each well of a UV‐transparent 96‐well plate were added 180 μL of a bovine erythrocyte GPx1 solution (0.125 U/mL) and 30 μL of prospective inhibitor in different concentrations diluted in DMF and incubated under shaking for 15 min at room temperature. Then 30 μL of stock solutions of GSH (2.5 mm) and baker's yeast GR plus NADPH (2 U/mL; 2.0 mm) were added. All stock solutions were prepared in potassium phosphate buffer (50 mm, pH 7.4, EDTA 1.1 mm, Triton‐X 0.01 %). The reaction was started by addition of TBHP solution (5 mm), giving final concentrations of 0.075 U/mL bovine erythrocyte GPx, 0.2 U/mL GR, 0.25 mm GSH, 0.2 mm NADPH and prospective inhibitor concentrations between 0.2–100 μm. The reaction was followed by the rate of decreasing absorption of the NADPH at *λ*=340 nm, measured every 15 s for ca. 20 min. The GPx activity related to untreated control was imported into the GraphPad Prism 6.0 software and the IC_50_ values were calculated by estimating the inflection point of the sigmoidal log concentration‐activity curves.

The jump‐dilution experiments were carried out in a 96‐well plates at room temperature. To each well was added 75 μL of a 20 IU mL^−1^ GPx stock‐solution followed by the addition of 3 μL of a 2.5 mm solution of **4** in DMF and 30 μL of a 2.5 mm solution of GSH in buffer or 30 μL of a 2.5 mm solution of GSH in buffer and 30 μL of a 5 mm solution of TBHP in water and 300 μL buffer. The plates were shaken for 30 min in the dark, then each well was diluted 100 fold into a second plate prepared for the GPx‐assay. The enzyme activity was immediately measured as described above.

To measure the inhibition of human GPx activity, the enzyme assay was adapted to use human cancer cell lysates in place of the bovine enzyme. A quantity of 100 μg cellular protein (determined according to the Bradford method) per well was used. Due to the large consumption of cell lysates, just the inhibitory effect on GPx at one concentration of pentathiepin was determined in primary screening. The concentration of GSH was raised to a concentration of 0.5 mm to increase the velocity of the reaction, all other conditions were unchanged. The rates of NADPH oxidation were normalized to the solvent control. For the measurement of the maximum velocity of GPx in cell lysates (*V*_max_), the activity of the cell lysates at various concentrations of glutathione were measured at 23 °C. We used 100 μg protein of lysates and added GSH up to final concentrations between 0.375–3 mm. The rates of the GPx activity were calculated by nonlinear regression and the fitted with the GraphPad Prism 6.0 software: “Enzyme kinetics – Substrate vs. Velocity (Michaelis‐Menten)”.

**Off‐target assays**: Possible inhibition of rat thioredoxin reductase and yeast GR was assessed as previously described.^33^ Inhibition of bovine catalase was determined by a previously reported method based on the reduction of dichromate to chromic acetate upon heating with hydrogen peroxide in acetic acid.^35^ Test tubes containing 3 mL of a 2 mg/l catalase solution in potassium phosphate buffer (50 mm, pH 7.4, 1.1 mm EDTA, 0.01 % Triton‐X) were incubated with or without pentathiepin for 5 min at 23 °C. To each sample was added 61.8 μL of 30 % hydrogen peroxide solution, then 1 mL was taken from each sample and mixed with 2 mL of a 3 : 1 mixture of glacial acetic acid and an aqueous 5 % solution of K_2_Cr_2_O_7_. These samples represent the H_2_O_2_ content at time zero. The remaining reaction mixtures were shaken for 10 min at 23 °C, then treated with the dichromate acid solution as described above. All samples were then heated in boiling water for 10 min and the absorption at *λ*=570 nm measured in a 1 cm cuvette with a Spectramax Plus 384 Reader. The inhibitory activity of pentathiepins was calculated by dividing the ΔA/Δt of the treated with the Δ*A*/Δ*t* of the untreated controls.

The superoxide dismutase assay is based on the ability of the enzyme to diminish the superoxide dependent reduction of nitro‐blue tetrazonium (NBT) to the blue formazan dye, whereby superoxide is generated by the xanthine/xanthine oxidase system as described previously.^36^ The reduction of NBT to a blue formazan was detected spectrophotometrically at *λ*=560 nm in presence or absence of SOD. For these studies, bovine SOD [Cu−Zn] was used, which is highly homologous to the human SOD [Cu−Zn] and commonly used in screening for SOD inhibitors. In each well of a 96‐well plate was added 300 μL sodium phosphate buffer (100 mm, pH 7.4) containing 1 μg/mL SOD, 100 μM xanthine, 1 mm NBT and 0.35 U/l xanthine‐oxidase in the presence or absence of pentathiepins. Controls without inhibitor represent 100 % SOD activity, controls without SOD represent 100 % inhibition. For the calculation of SOD inhibitory effect, SOD activity measured in the presence of pentathiepin was divided by the SOD activity without pentathiepin.

**Cell culture**: The human acute myeloid leukemia cell line HL‐60 and human cervical cancer cell line SISO were obtained from Deutsche Sammlung von Mikroorganismen und Zellkultur, DSMZ (Braunschweig, Germany), the Burkitt lymphoma cell line Gumbus was provided by Prof. G. Dölken (Universitätsmedizin Greifswald, Germany) and the human chronic myeloid leukemia cell line HAP‐1 cell was purchased from Horizon Discovery (Cambridge, UK). All cell lines were routinely tested for mycoplasma. Gumbus, HL‐60 and SISO cells were grown in RPMI 1640 supplemented with 10 % FBS and 1 % penicillin/streptomycin while the HAP‐1 cells were cultured in IMDM medium supplemented with 1 % stable glutamine, 10 % FBS and 1 % penicillin/streptomycin. Cells were cultured at 37 °C in a humidified incubator with 5 % CO_2_ atmosphere. Detachment with trypsin/EDTA was used to harvest the adherent cell lines SISO and HAP‐1.

**Crystal violet proliferation assay**: To measure cell growth inhibition, the crystal violet assay was carried out as previously described.^37^ Adherent cell lines were seeded out in a 96‐well plate (1000 cells per 200 μL culture medium) and allowed to attach for 24 h. Cells were exposed to nine serial dilutions of compound, added to the medium from 1000‐fold concentrated stock solutions in DMF. For the controls, cells were exposed to 0.1 % DMF alone. After addition of compounds, plates for the detection of the starting point of the proliferation at incubation time zero (*t*_0_) were stopped with glutaraldehyde (see below), washed with Dulbecco's buffer (containing KCl 0.2 g/L, MgSO_4_ ⋅ 7 H_2_O 0.1 g/L, Na_2_HPO_4_ ⋅ **>**7 H_2_O 1.55 g/L, KH_2_HPO_4_ 0.2 g/L and NaCl 8 g/L in water) and stored at 4 °C until the staining procedure. Treated cells were incubated for 96 h in the incubator, the medium was removed and replaced with 100 μL of 1 % glutaraldehyde in Dulbecco's buffer for 20 min. After washing twice with 150 μL of Dulbecco's buffer, the buffer was removed and replaced with 100 μL of a 0.02 % crystal violet solution dissolved in water for 30 min and subsequently washed in tap water for 15 min. Water was removed and replaced with 100 μL of ethanol (70 % in water) followed by shaking on a 96‐well shaker (MS3 digital, IKA‐Werke, Germany) for 2 h. Optical density was measured with a SpectraMax Plus 383 microtiter plate reader at *λ*=570 nm (Molecular Devices, USA). The optical density of *t*_0_ plates at the time of treatment were subtracted from optical densities of plates with treated cells after 96 h (T), and the growth inhibition relative to untreated, control cells (C) as described.^22^ Growth inhibition concentrations at 50 % (GI_50_) were calculated by determining the inflection point of the sigmoidal log dose‐growth inhibition (%T/C) curves with the GraphPad Prism 6.0 software.

**MTT cell viability assay**: For the detection of the inhibition of the viability of cells by the pentathiepins, the MTT‐viability assay was used.^37^ Briefly, 10 000 and 20 000 cells for the suspension cell lines HL‐60 and Gumbus, respectively, were seeded out in 50 μL medium per well and immediately exposed to the compounds. For the adherent cell lines SISO and HAP‐1, 3000 and 5000 cells, respectively, were seeded out in 96‐well plates and allowed to attach for 24 h. Cells were exposed to nine serial dilutions of compound, added to the medium from 1000‐fold concentrated stock solution in DMF. For the controls, cells were exposed to 0.1 % DMF alone. After 48 h incubation, 20 μL of the MTT stock solution (2.5 mg/mL in PBS) were added to each well to give a final concentration of 1.0 mm. The formazan crystals that formed after 4 h at 37 °C were dissolved by sonification after adding 100 μL of 0.04 M HCl in isopropanol for the suspension cell lines, or with 50 μL DMSO after aspiration of the medium for the adherent cell lines. Optical density was measured with a SpectraMax Plus 383 microtiter plate read at *λ*=570 nm (Molecular Devices, USA). Data of related absorption to control cells were imported into GraphPad Prism 6.0 software and IC_50_ values were generated by determining the inflection point of the sigmoidal log concentration‐viability inhibition curves.

**Detection of reactive oxygen species**: To detect cellular reactive oxygen species (ROS) a flow cytometry technique based on the use of the ROS‐sensor 2′,7′‐dichlorofluorescin diacetate (DCF‐DA) was used.^38^ Briefly, cells were collected in reaction tubes and washed with 500 μL phosphate buffered saline (PBS). The cell pellets were resuspended in 1 mL PBS and stained with 2 μL DCF‐DA stock solution (10 mm in DMSO) for 30 min at 37 °C. Then cells were washed with 500 μL PBS and suspended in 1 mL PBS followed by addition of 1 μL **4** (50 mm in DMF) to a final concentration of 25 μM. After an incubation period of 10 min cells were washed with PBS, resuspended in 500 μL PBS and analyzed by flow cytometry with a MACSQuant Analyzer 10 (Miltenyi Biotec, Germany) set at *λ*_Ex/Em_=488/530 nm.

**Fluorescence microscopy of mitochondrial membrane potential**: To detect possible effects of **5** on the mitochondrial membrane potential (MMP) of HAP‐1 cells the fluorescent dye JC‐1 was used.^39^ JC‐1 accumulates into living cells and is present in monomers in the cytosol. It can also cross the membrane of active mitochondria, were aggregates form. When excited at *λ*=488 nm, JC‐1 monomers give a green fluorescence while JC‐1 aggregates show red fluorescence. As a positive control, an inhibitor of the oxidative phosphorylation, the fluorescent carbonyl cyanide‐*p*‐trifluoromethoxyphenylhydrazone (FCCP), was used. Briefly, 10^5^ cells in 500 μL medium were seeded out into a chamber of a four‐well chamber‐slide and allowed to attach for 24 h. After incubation with **4** at various concentrations, cells were exposed to the JC‐1 solution for 15 min according to the manufacturer's protocol (BD Bioscience, USA). Cells on the slides were then mounted and examined with a Leica DMi8 fluorescence microscope (Leica, Germany), at *λ*_ex/em_=488/512–542 nm for JC‐1 monomers and *λ*_ex/em_=488/565–605 nm for agglomerates.

**Fluorescence microscopy for the detection of lipid peroxidation**: SISO cells (250 000 cells) were seeded out in 6‐well plates with an inserted coverslip. Cells were allow to attach for 24 h and then exposed to 25 μM **4** or 100 μM *tert*‐butyl‐hydroperoxide as a positive control. After 24 h coverslips were washed with PBS and stained with 1 μM BODIPY 581/591 in culture medium for 30 min. Cells on the coverslips were then mounted and examined with a Leica DMi8 fluorescence microscope at *λ*_ex/em_=460–500/512–542 nm for the oxidized BODIPY 581/591 and *λ*_ex/em_=540–581/592–668 for the reduced form.

**Cleavage of supercoiled plasmid DNA**: For this assay, 0.3 μg pBR322 plasmid (Thermo Fisher) were incubated with either 0.1 % DMF or 5 μM **4** with or without 2 mm glutathione (GSH) in a 50 mm sodium phosphate buffer at pH 7.5 for 20 h in a 37 °C water bath. Afterwards, the samples were separated on a 1 % agarose (Geneon) gel at 80 V (5 V/cm) for 2 h, stained with GelRed (Biotium) and the image captured and bands quantified using ImageLab (Biorad). The resulting data was analyzed and visualized with Prism 7 (GraphPad).

**Alkaline comet assay to assess induction of DNA strand breaks**: The method was adapted from Olive and Banath.^40^ The alkaline protocol allows for the detection of single and double strand breaks as well as alkali‐labile sites and is based on the different electrophoretic mobility of intact and damaged nuclear DNA. During electrophoresis intact genomic DNA has a low electrophoretic mobility and does not migrate in the gel, which results in the formation of the comet head. The induction of strand breaks creates free charged ends that drift out and form the characteristic comet tail. The percentage of DNA that is located in the comet head was selected as descriptor, which corresponds to the amount of still intact genomic DNA.

Briefly, SISO and HAP‐1 cells were harvested by trypsinization and a single cell suspension with 50 000 cells per mL PBS was prepared. Per condition 50 000 cells were incubated on ice (to impair DNA repair) with either the solvent control DMF (1.0 %), 20 μM H_2_O_2_ or 25 μM **4** for 15 min. Afterwards, 400 μL of the cell suspension were mixed with 1.2 mL 1 % low melting point agarose (Carl Roth, Germany) at 40 °C and evenly distributed on agarose‐precoated (GeneOn, Germany) glass slides (Thermo Fisher). After 3 min polymerization the slides were horizontally submerged in lysis buffer (1.2 M NaCl (Carl Roth, Germany), 0.1 % N‐Lauryl‐Sarcosinate (Merck), 0.1 M Na‐EDTA, 0.26 M NaOH) and placed at 4 °C overnight. Before electrophoresis at 0.6 V/cm for 25 min the slides were rinsed three times in rinse/electrophoresis buffer (0.002 M Na‐EDTA, 0.03 M NaOH). Afterwards, the slides were neutralized in distilled water and subsequently stained with 250 μL of a 10 μg/mL PI in water solution for 20 min. Finally, the slides were analyzed with a Leica DMi8 fluorescent microscope and LASX software (Leica, Germany) and comets were scored with CometScore 2.0 (Rex A. Hoover, www.rexhoover.com). At least 100 comets were scored per condition.

**Apoptosis staining with Annexin‐V/PI**: For the apoptosis measurement, the translocation of phosphatidyl serine from the inner to the outer membrane by Annexin‐V and PI double staining was used.^41^ The protocol of the manufacturer (Miltenyi Biotec, Germany) of the “Annexin‐V‐FITC Apoptosis Detection Kit” was followed. In brief, 500,000 cells were seeded into 6‐well plates and exposed to **4** at multiple concentrations of the viability IC_50_ for 6 and 24 h. Afterwards, the cells were collected in reaction tubes, washed with the binding buffer belonging to the Annexin‐V‐Kit and stained with Annexin‐V‐FITC for 10 min. After repeated washing steps, the PI solution was added to the cells and followed by the flow cytometry investigation analyses at *λ*_ex/em_=488/525±50 nm for FITC and *λ*_ex/em_=488/655–730 nm for PI with a MACSQuant Analyzer 10 (Miltenyi Biotec).

**Western blot analysis of PARP**: For western blot analysis the Biorad system with “Mini‐Protean® TGX Stain‐Fee™ Gels” and “Trans‐Blot® Turbo™ Transfer Pack Mini” PVDF‐membranes were used (Biorad, Germany). Protein lysates for analysis were prepared by seeding out 10^6^ cells into T_25_ flasks and incubated with **4** in multiple concentrations of the viability IC_50_ for 6 and 24 h. After harvesting and washing cells with PBS, the cells were lysed with a lysis buffer containing Tris 50 mm (pH 7.4), 100 mm NaCl, 100 mm NaF, 5 mm EDTA, 0.2 mM Na_3_VO_4_, 0.1 % Triton‐X and freshly added 1 % protein inhibitor cocktail (Sigma Aldrich), on ice for 30 min followed by a sonification for 10 min. Lysates were centrifuged at 18 000 *g* for 10 min at 4 °C and protein content was determined by the Bradford method against bovine serum albumin (BSA) as standard. To each slot Preotean® gels were loaded 30 μg of protein, followed by electrophoretic separation and transfer onto PVDF‐membranes. Before incubation with antibody, the blots were blocked with 10 % non‐fat milk powder in Tris buffered saline (containing 2.42 g/l Tris, 8.48 g/l NaCl in water) plus tween (0.5 %; TBST). The blots were incubated over night with PARP‐antibody (1 : 1000 dilution in TBST plus 1 % BSA) at 4 °C before the secondary antibody from rabbit (1 : 10 000 in TBST plus 1 % BSA), conjugated with horse radish peroxidase, was incubated for 1 h at room temperature. Protein bands were detected with Clarity™ Western ECL Substrate (Biorad) and recorded by an Advanced Fluorescence Imager (INTAS, Germany).

**Statistical analysis**: The results of at least three independent experiments were expressed as the means with standard deviations (SD). Unless otherwise stated, statistical significance was determined by using the analysis of variance (ANOVA) followed by a multiple comparison with a Dunnett's test. The statistical analysis was performed with the GraphPad Prism 6.0 or 7.0 software. Statistical significance was expressed by *p<0.05, **p<0.01, ***p<0.001 or ****p<0.0001.

## Conflict of interest

The authors declare no conflict of interest.

## Supporting information

As a service to our authors and readers, this journal provides supporting information supplied by the authors. Such materials are peer reviewed and may be re‐organized for online delivery, but are not copy‐edited or typeset. Technical support issues arising from supporting information (other than missing files) should be addressed to the authors.

Supplementary
