# Supplementary material for: Pentathiepins: A Novel Class of Glutathione Peroxidase 1 Inhibitors that Induce Oxidative Stress, Loss of Mitochondrial Membrane Potential and Apoptosis in Human Cancer Cells
Source: ChemMedChem. 2020 May 6;15(16):1515–28. doi: 10.1002/cmdc.202000160 (PMC7496275; doi:10.1002/cmdc.202000160)
Supplement: Supplementary file 1 — Supplementary [file CMDC-15-1515-s001.pdf]

ChemMedChem

Supporting Information

Pentathiepins: A Novel Class of Glutathione Peroxidase 1 Inhibitors that Induce Oxidative Stress, Loss of Mitochondrial Membrane Potential and Apoptosis in Human Cancer Cells

Steven Behnisch-Cornwell, Siva Sankar Murthy Bandaru, Martin Napierkowski, Lisa Wolff, Muhammad Zubair, Claudia Urbainsky, Christopher Lillig, Carola Schulzke, and Patrick J. Bednarski*© 2020 The Authors. Published by Wiley-VCH Verlag GmbH & Co. KGaA. This is an open access article under the terms of the Creative Commons Attribution License, which permits use, distribution and reproduction in any medium, provided the original work is properly cited.

Supporting Information

Compounds 1-5

General

The compounds **1** and **4** were synthesized as reported previously in the protocol from Zubair et.al [1]. They had purities of ≥ 95% as indicated by HPLC (detection @ λ = 250 nm). Compounds **2, 3** and **5** were synthesized here for the first time with the same protocol.

All the reactions were performed under nitrogen atmosphere using oven dried standard Schlenk glassware. The completely dried *N,N* dimethylformamide (DMF, 99.8%, extra dry, stored over molecular sieves) was purchased from Acros organics and used as received for all air or moisture sensitive reactions. ^{1}H NMR (300 MHz) and ^{13}C NMR (100 MHz) spectra were recorded on a Bruker Avance II-300 spectrometer. Chemical shifts δ are given in ppm and the solvent residual peak ($CDCl_3$: ^{1}H, δ = 7.27; ^{13}C, δ = 77.0 and DMSO-d6: ^{1}H, δ = 2.50; ^{13}C, δ = 40) was used as an internal standard. Peak multiplicities are specified as followed: s, singlet; d, doublet; t, triplet; q, quartet; m, multiplet; br, broad. APCI-MS (m/z) spectra were recorded on an Advion MS. Macherey-Nagel silica gel 60 F254 plates were used for thin layer chromatography (TLC) and detection was achieved by UV light. Column chromatography was performed on silica gel 60 (40-63 μm) or on Acros Organics silica gel 60 (35-70 μm). The X-ray single crystal structure experiments were conducted with a "STOE IPDS2T" diffractometer equipped with a fine- focus sealed molybdenum tube. The "Elementar Vario MICRO cube" was used for the experimental determination of elemental configurations of final pure products. Melting points (uncorrected) were determined with a Büchi 545 (Büchi, Flawil, CH).

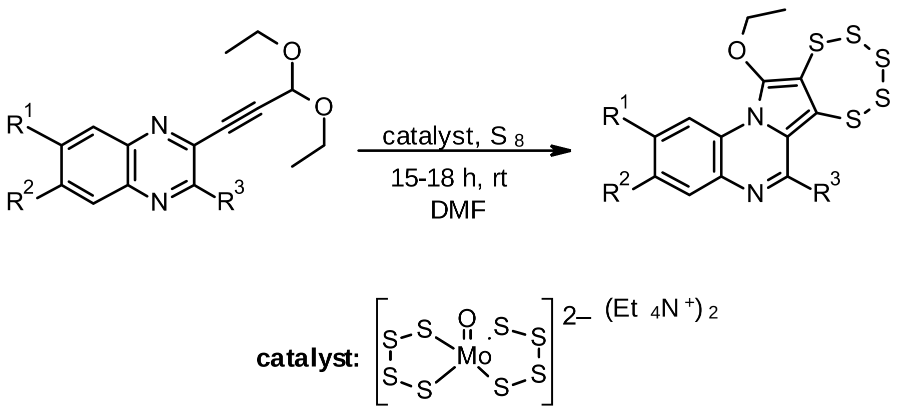

Scheme S1. Synthesis of pentathiepins **1-5** mediated by the Mo-catalyst.

HPLC of compounds 1 and 4

RP-HPLC were performed with a Merck-Hitachi LaChrom 7000 instrument fitted with a Merck Chromolith SpeedROD RP-18e column (4.6 x 50 mm) and held at 30 °C. Samples of 25 µL were injected and eluted with a solvent of 80% acetonitrile/water at a flow rate of 1.0 mL/min. Detection was done between the wavelengths of 210 and 500 nm.

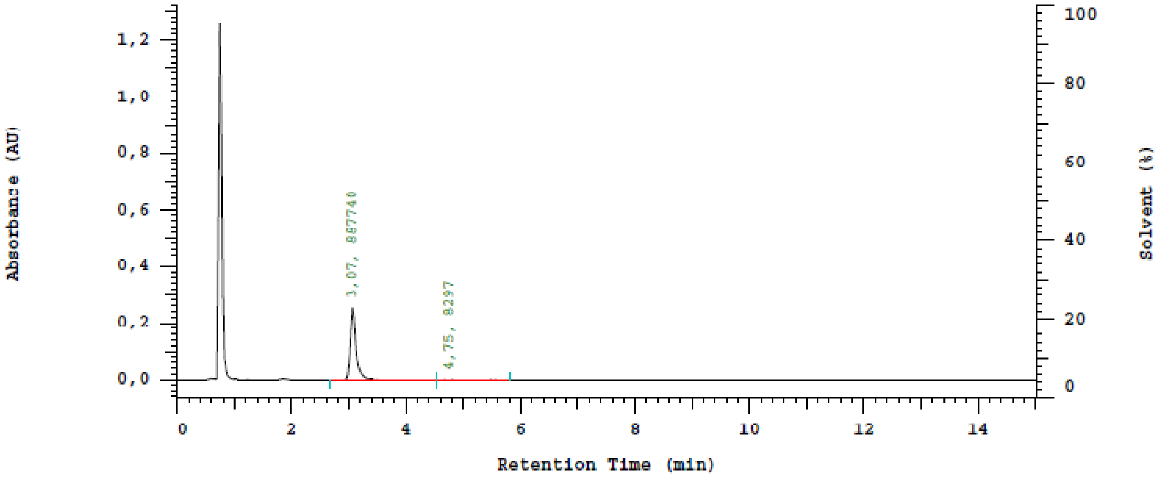

Figure S1. RP-HPLC of compound **1**, rt = 3.07 min, detected at λ = 250 nm. Peak at 0.78 min is from DMF.

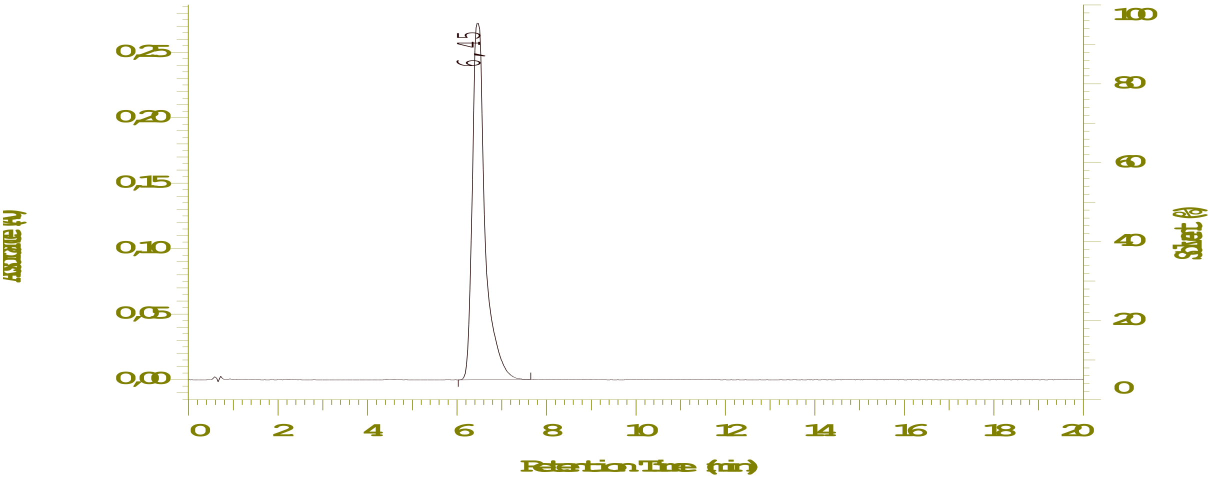

Figure S2. RP-HPLC of compound **4**, rt = 6.45 min, detected at λ = 250 nm.

^{1}H, ^{13}C and ^{19}F NMR, APCI-MS spectra and HPLC of 2,3 and 5

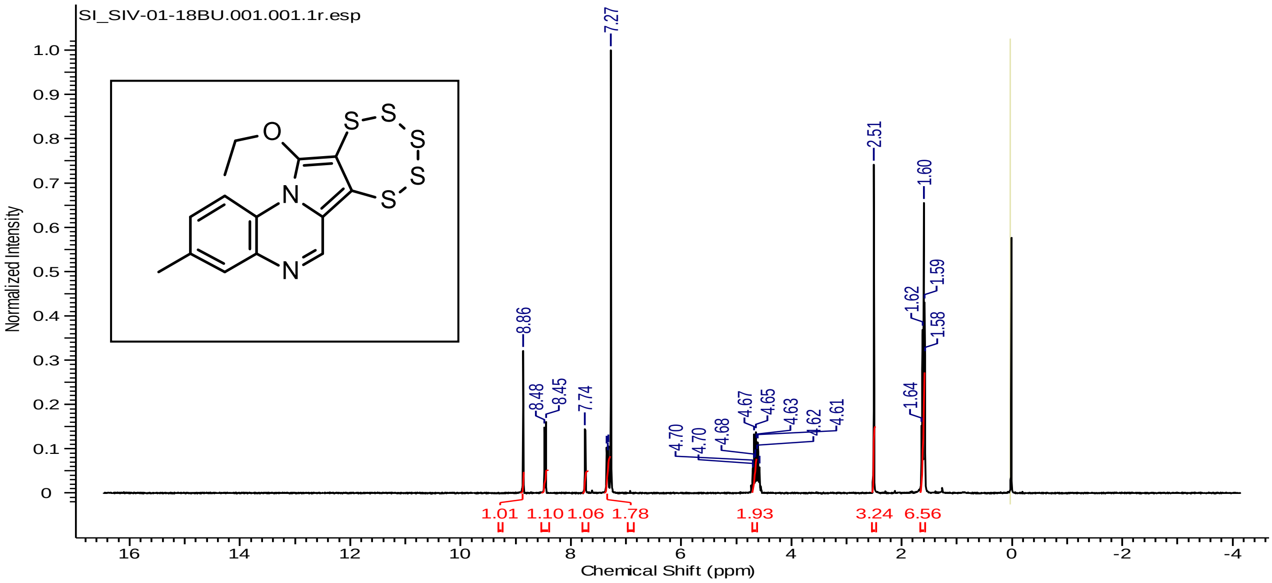

Figure S3. ^{1}H-NMR ($CDCl_3$) of compound **2**.

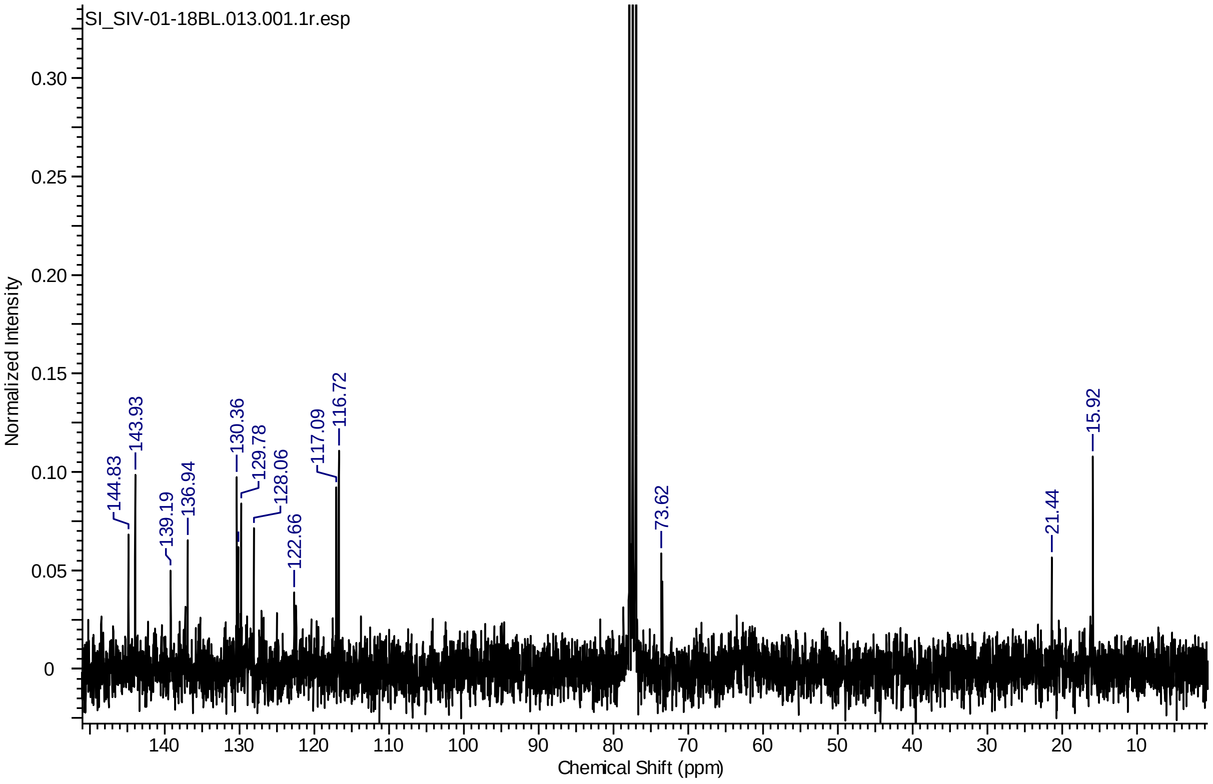

Figure S4. ^{13}C-NMR ($CDCl_3$) of compound **2**.

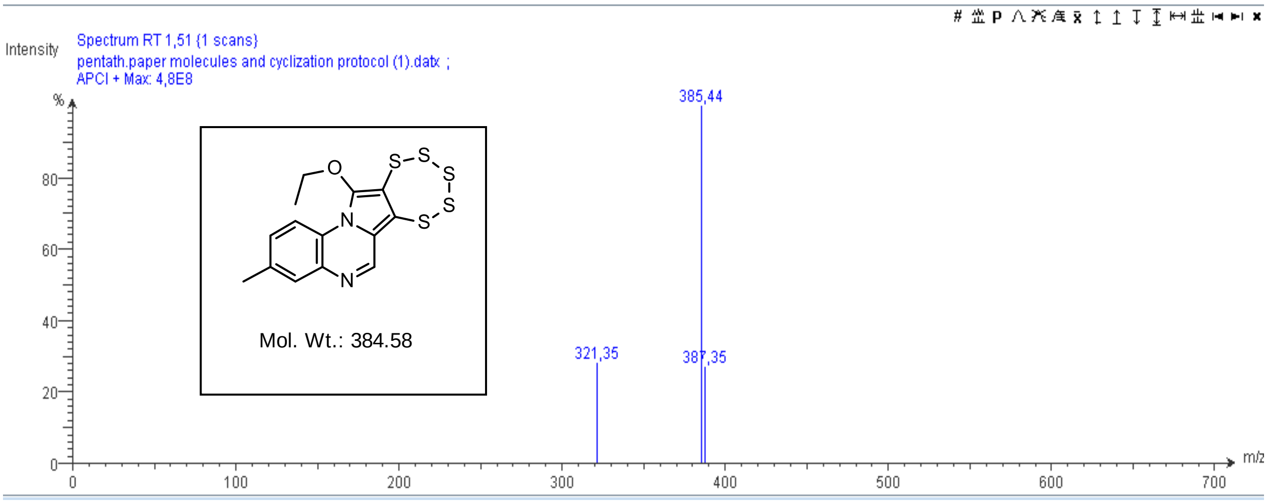

Figure S5. APCI-MS spectrum for compound **2**.

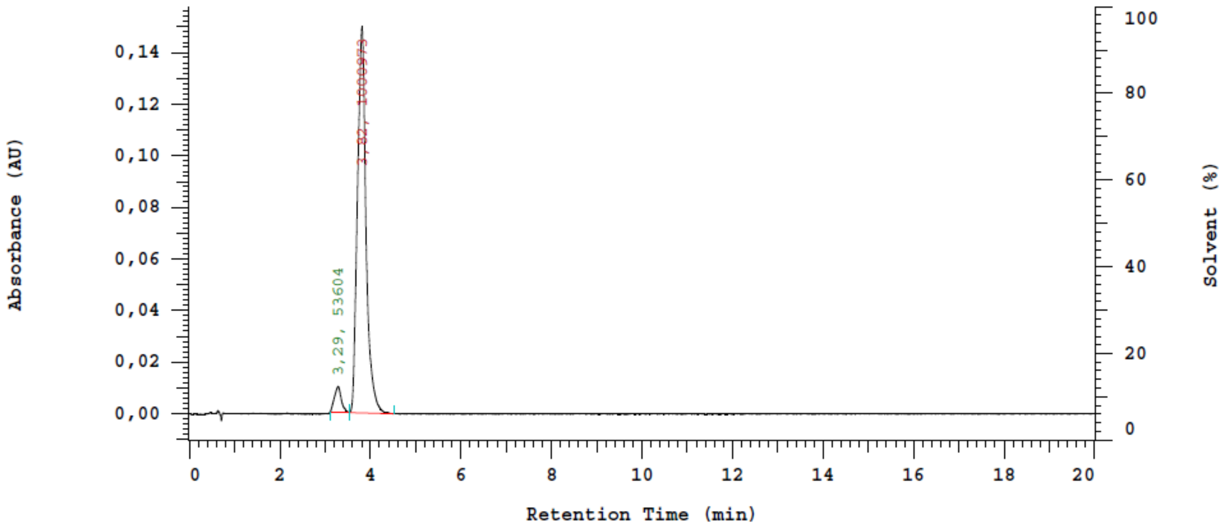

Figure S6. RP-HPLC of compound **2**, rt= 3.82 min, detected at λ = 250 nm.

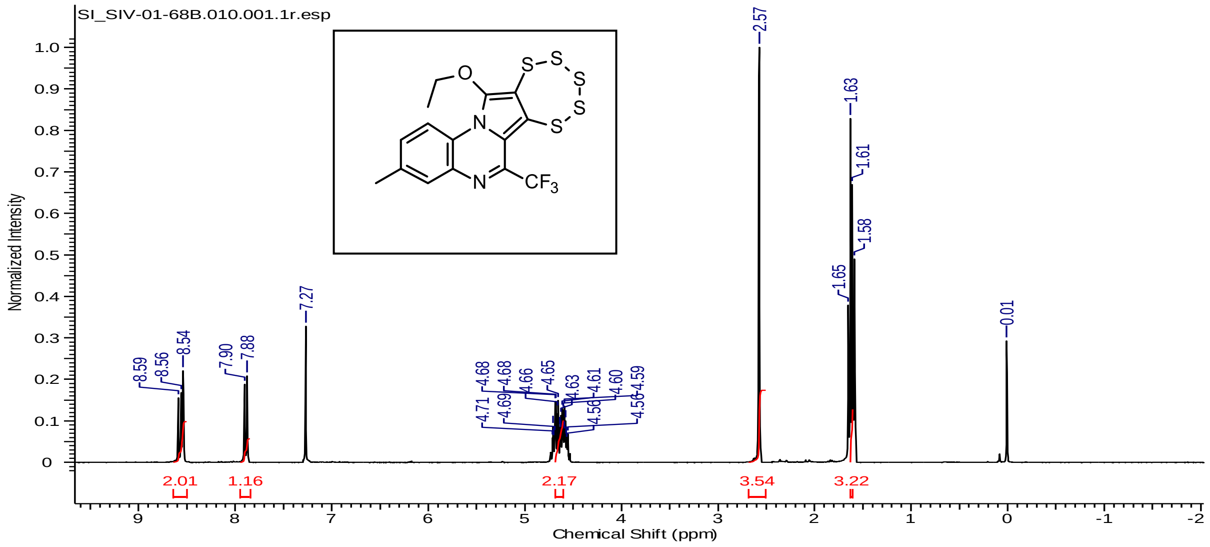

Figure S7. ^{1}H-NMR ($CDCl_3$) of compound **3**.

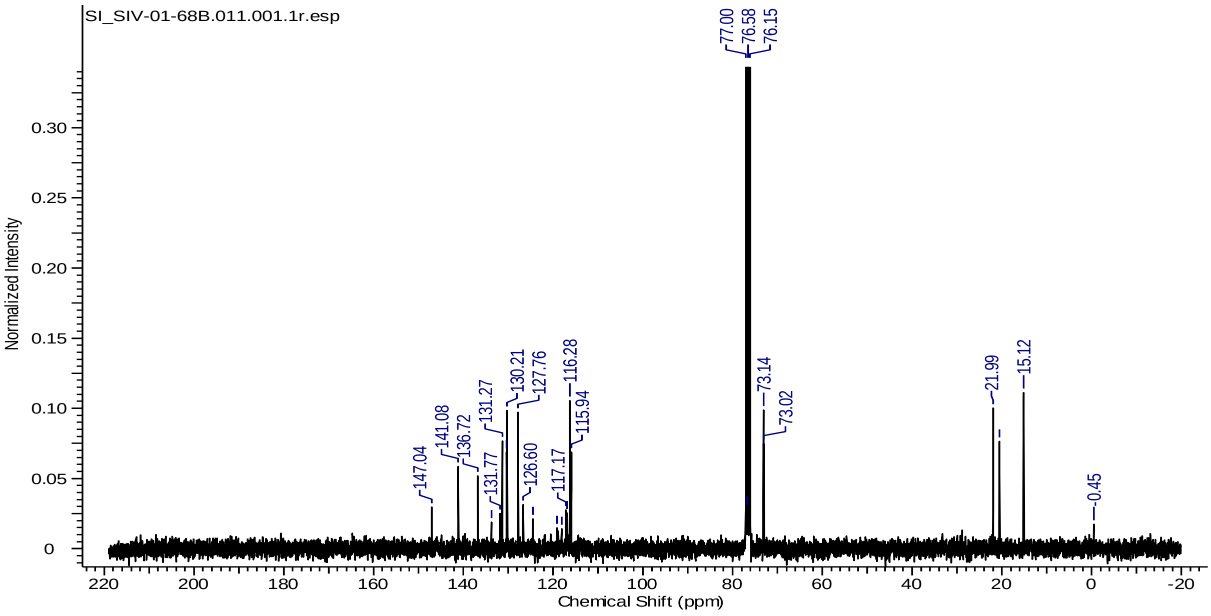

Figure S8. ^{13}C-NMR ($CDCl_3$) of compound **3**.

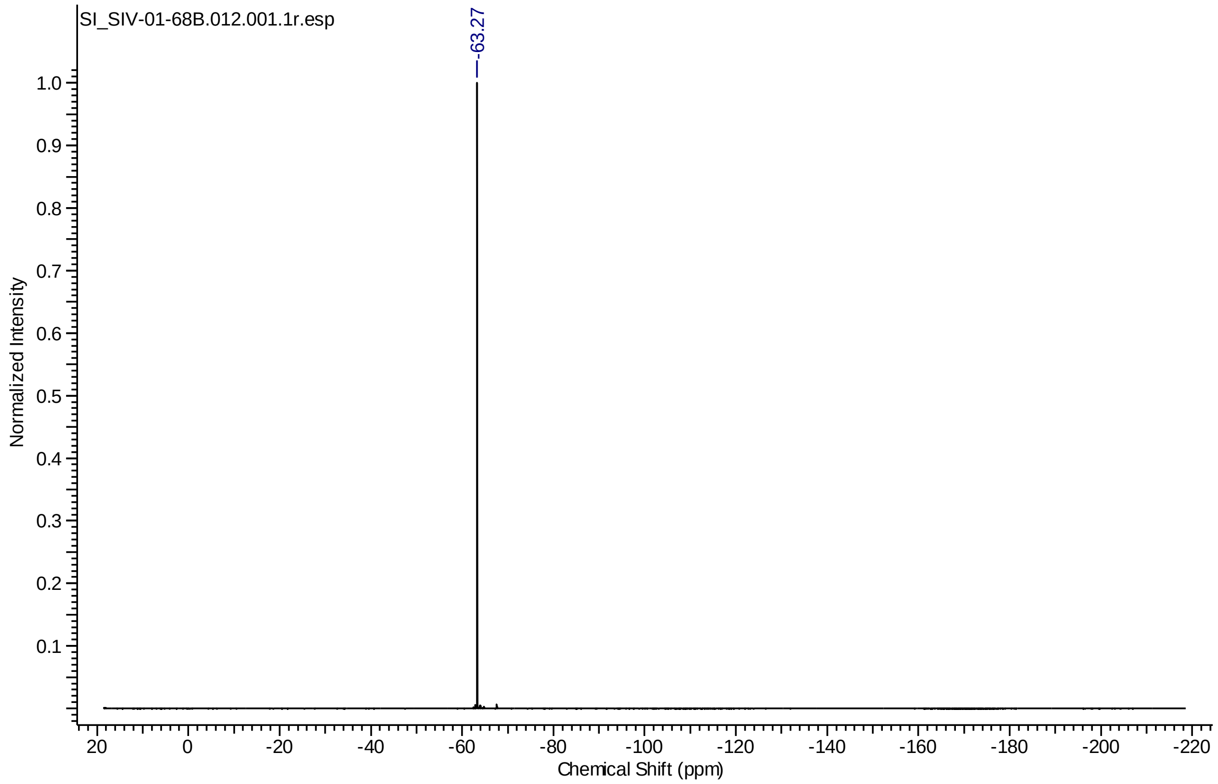

Figure S9. ^{19}F-NMR ($CDCl_3$) of compound **3**.

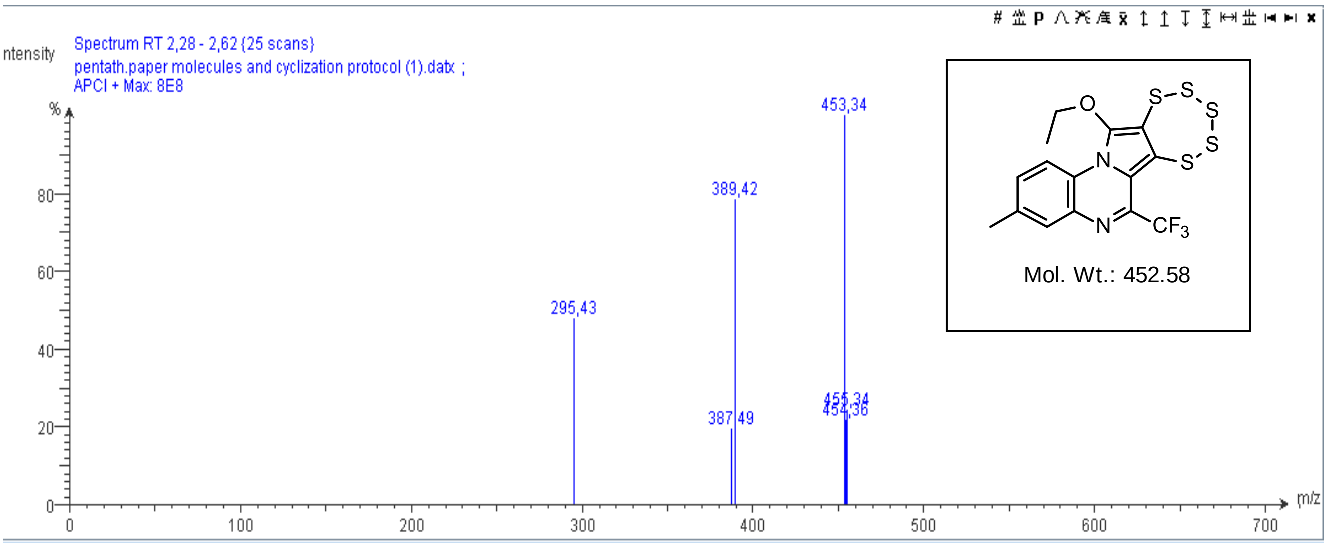

Figure S10. APCI-MS spectrum for compound **3**.

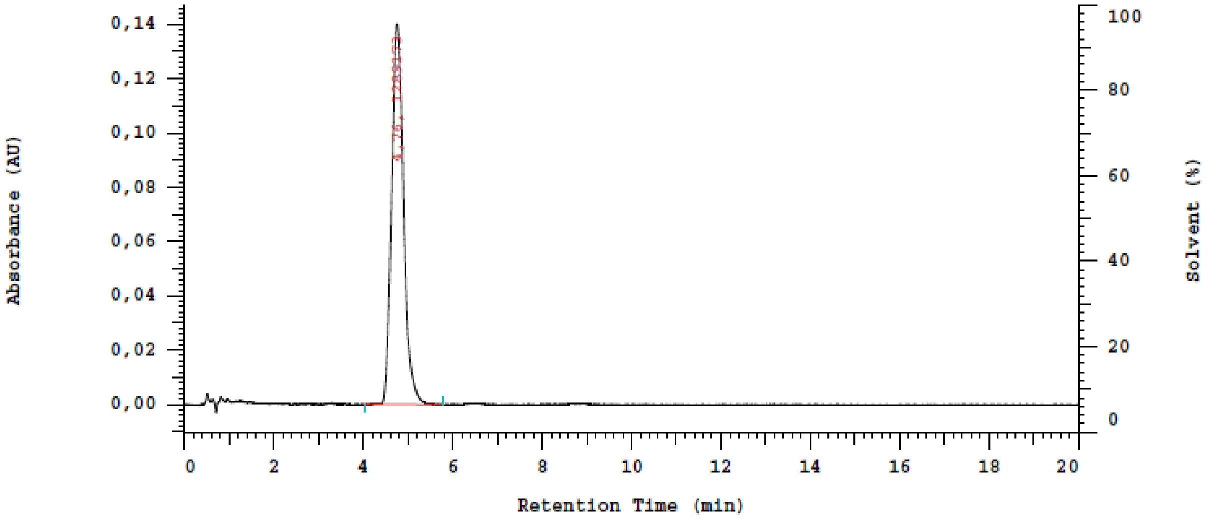

Figure S11. RP-HPLC of compound **3**, rt = 4.76 min, detected at λ = 250 nm.

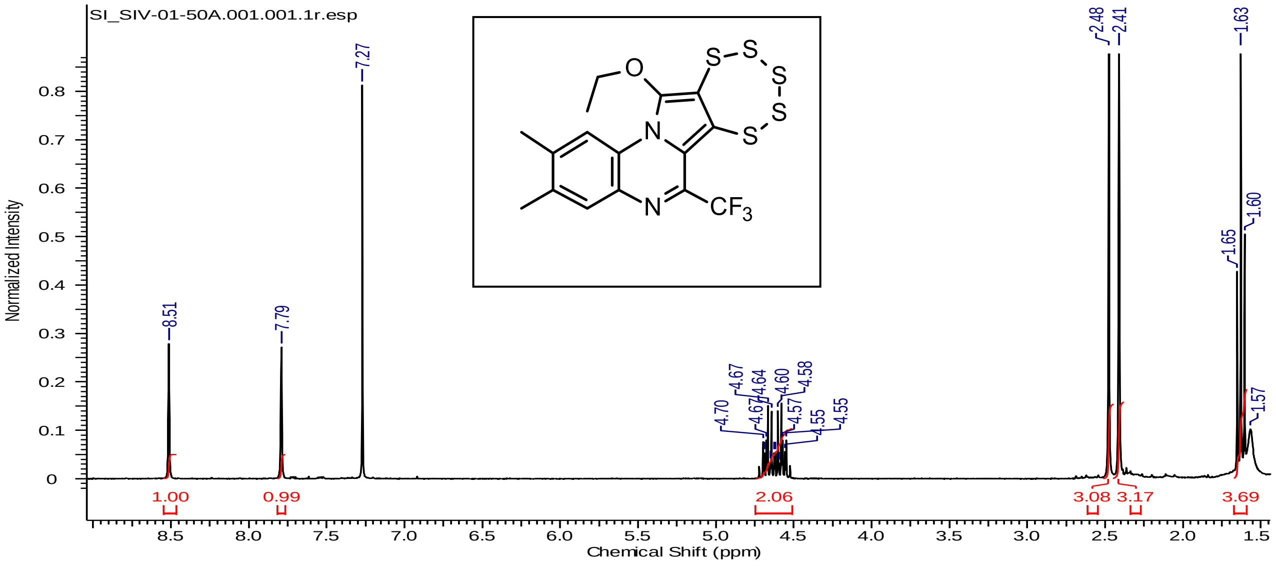

Figure S12. ^{1}H-NMR ($CDCl_3$) of compound **5**.

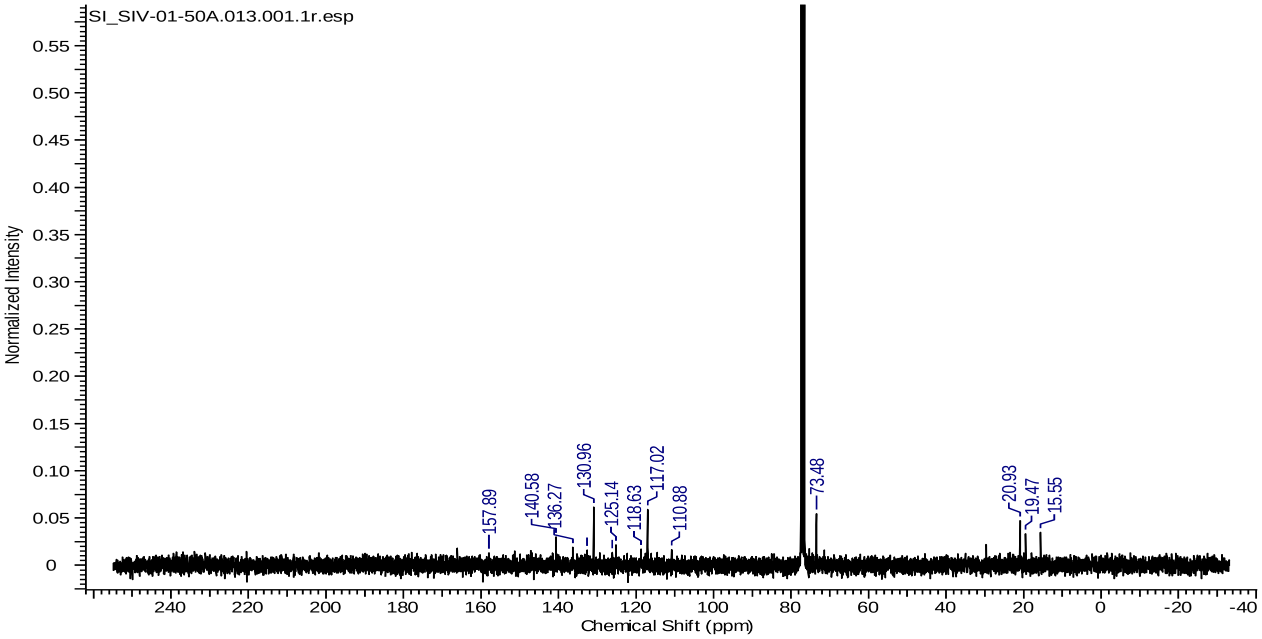

(Note: intensity of peaks were very low as the concentration of sample was diluted. Overnight measurement was not available)

Figure S13. ^{13}C-NMR ($CDCl_3$) of compound **5**.

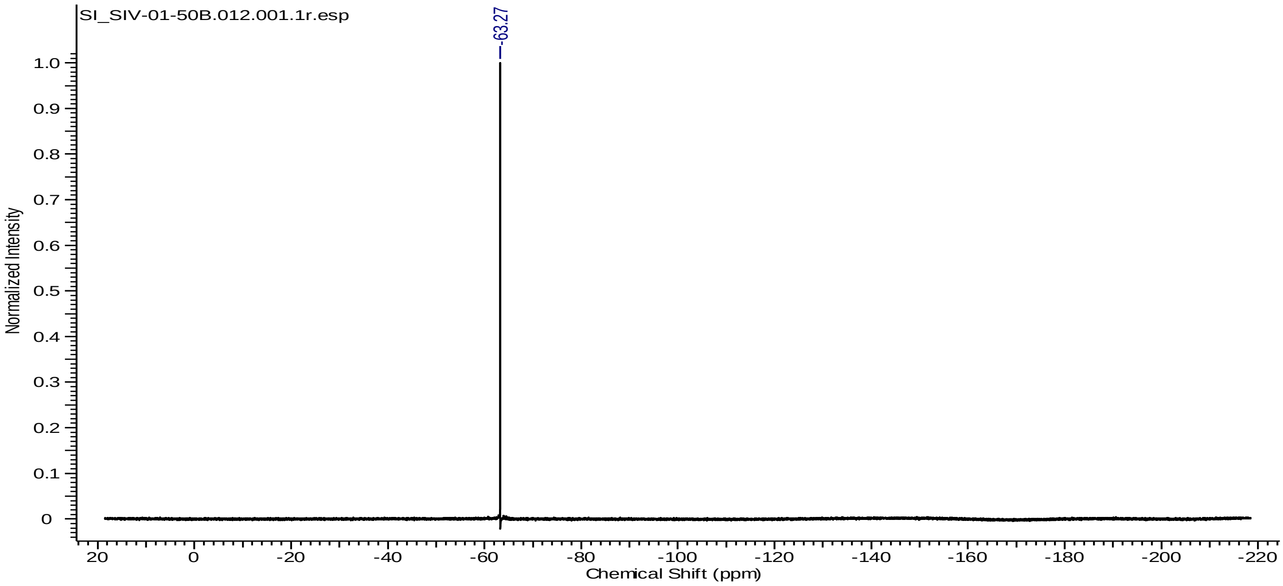

Figure S14. ^{19}F-NMR ($CDCl_3$) of compound **5**.

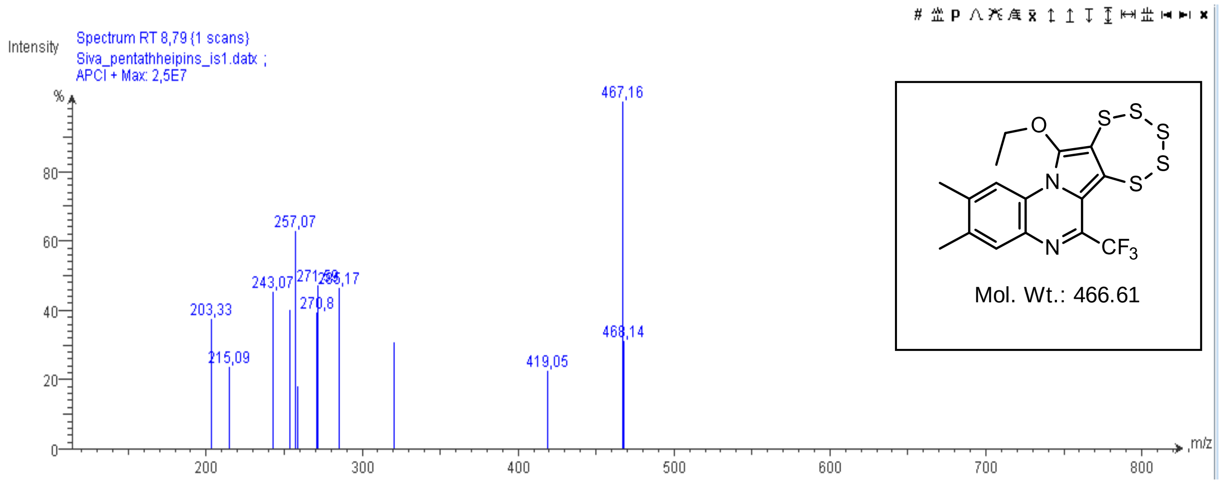

Figure S15. APCI-MS spectrum for compound **5.**

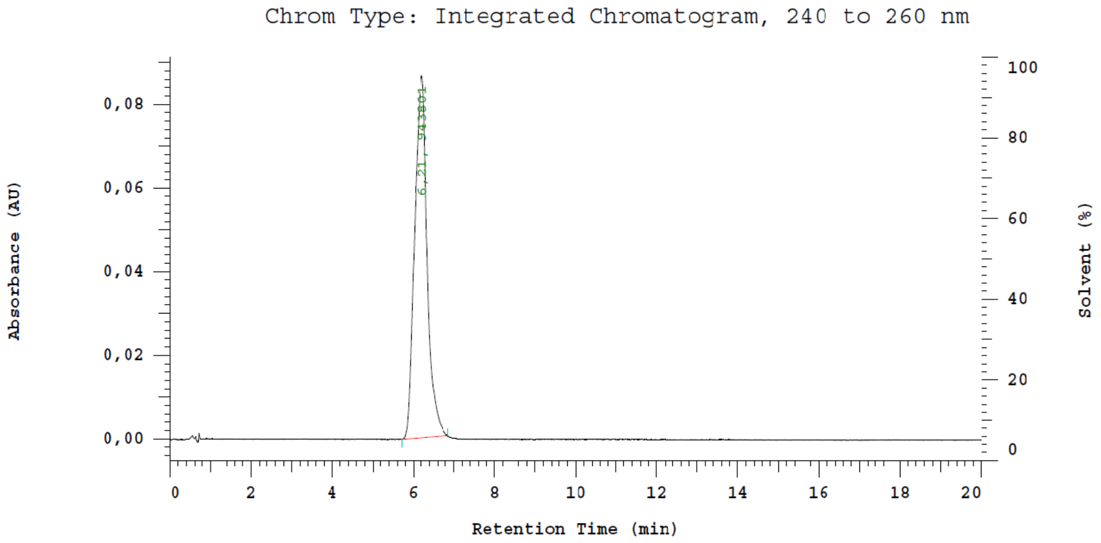

Figure S16. RP-HPLC of compound **5**, rt = 6.21 min.

X-ray Crystallographic data for 5

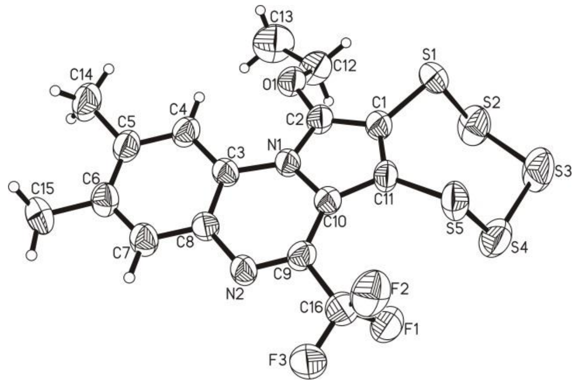

Figure S17. X-Ray crystal structure for compound **5.**

Table S1. Crystal data and structure refinement for **5**.

Identification code	SIV-01-50A
Empirical formula	$C_{16}H_{13}F_3N_2O_1S_5$
Formula weight	466.58
Temperature	298(2) K
Wavelength	0.71073 A
Crystal system, space group	Monoclinic, $P2_1/c$
Unit cell dimensions	a = 18.941(4) A alpha = 90°
	b = 11.048(2) A beta = 94.92(3)°
	c = 9.2081(18) A gamma = 90°
Volume	1919.8(7) $Å^3$
Z, Calculated density	4, 1.614 g/cm^3
Absorption coefficient	0.641 mm^{-1}
F(000)	952
Crystal size	0.468 x 0.076 x 0.053 mm
Theta range for data collection	1.079° to 26.128°
Limiting indices	-23<=h<=23, -13<=k<=13, -11<=l<=9
Reflections collected / unique	18211 / 18211 [R(int) = not determined due to hklf5 refinement]
Completeness to theta = 25.242	100.0 %
Absorption correction	Numerical
Max. and min. transmission	0.9859 and 0.7121
Refinement method	Full-matrix least-squares on F^2
Data / restraints / parameters	18211 / 0 / 248
Goodness-of-fit on F^2	1.223
Final R indices [I>2sigma(I)]	R1 = 0.1880, wR2 = 0.3928
R indices (all data)	R1 = 0.3032, wR2 = 0.4632
Extinction coefficient	0.011(6)
Largest diff. peak and hole	2.909 and -0.984 $e·Å^{-3}$

Table 2. Atomic coordinates (x 10^4) and equivalent isotropic displacement parameters (A^2 x 10^3) for siv0150a.
U(eq) is defined as one third of the trace of the orthogonalized Uij tensor.

	x	y	z	U(eq)

C(1)	1878(10)	2508(17)	1970(20)	48(5)
C(2)	2256(11)	1629(14)	1300(20)	49(5)
C(3)	3290(10)	1658(16)	-270(20)	42(5)
C(4)	3458(10)	418(16)	-230(30)	50(5)
C(5)	3990(11)	4(17)	-1060(20)	52(5)
C(6)	4337(10)	776(17)	-1940(30)	51(5)
C(7)	4170(10)	1990(16)	-1940(20)	51(5)
C(8)	3657(10)	2457(17)	-1090(20)	47(5)
C(9)	3091(10)	4137(16)	-270(20)	45(5)
C(10)	2677(10)	3435(15)	630(20)	43(5)
C(11)	2148(10)	3655(15)	1570(20)	45(5)
C(12)	1545(11)	-163(17)	1180(30)	65(7)
C(13)	1630(15)	-1450(20)	790(40)	94(9)
C(14)	4176(13)	-1327(18)	-960(30)	71(7)
C(15)	4905(12)	330(20)	-2860(30)	73(7)
C(16)	3017(12)	5514(17)	-380(30)	55(5)
F(1)	2344(7)	5857(10)	-727(15)	66(3)
F(2)	3229(8)	6052(11)	858(18)	76(4)
F(3)	3391(8)	5963(10)	-1406(17)	79(5)
N(1)	2754(8)	2176(12)	517(18)	42(4)
N(2)	3540(8)	3702(13)	-1130(20)	48(4)
O(1)	2226(7)	411(10)	1323(17)	54(4)
S(1)	1251(3)	2262(5)	3208(7)	58(2)
S(2)	314(3)	2746(6)	2041(9)	77(2)
S(3)	219(3)	4556(6)	2424(10)	88(3)
S(4)	925(4)	5347(5)	1146(9)	78(2)
S(5)	1895(3)	5038(5)	2242(7)	60(2)

Table 3. Bond lengths [A] and angles [deg] for siv0150a.

C(1)-C(2)	1.38(3)
C(1)-C(11)	1.43(3)
C(1)-S(1)	1.737(18)
C(2)-O(1)	1.348(19)
C(2)-N(1)	1.38(2)
C(3)-C(8)	1.39(2)
C(3)-C(4)	1.41(2)
C(3)-N(1)	1.42(2)
C(4)-C(5)	1.39(3)
C(4)-H(4)	0.9300
C(5)-C(6)	1.38(3)
C(5)-C(14)	1.51(3)
C(6)-C(7)	1.38(2)
C(6)-C(15)	1.51(3)
C(7)-C(8)	1.40(3)
C(7)-H(7)	0.9300
C(8)-N(2)	1.39(2)
C(9)-N(2)	1.30(2)
C(9)-C(10)	1.42(3)
C(9)-C(16)	1.53(3)
C(10)-C(11)	1.40(3)
C(10)-N(1)	1.40(2)
C(11)-S(5)	1.730(17)
C(12)-O(1)	1.43(2)
C(12)-C(13)	1.48(3)
C(12)-H(12A)	0.9700
C(12)-H(12B)	0.9700
C(13)-H(13A)	0.9600
C(13)-H(13B)	0.9600

C(13)-H(13C)	0.9600
C(14)-H(14A)	0.9600
C(14)-H(14B)	0.9600
C(14)-H(14C)	0.9600
C(15)-H(15A)	0.9600
C(15)-H(15B)	0.9600
C(15)-H(15C)	0.9600
C(16)-F(2)	1.31(3)
C(16)-F(3)	1.33(2)
C(16)-F(1)	1.34(2)
S(1)-S(2)	2.066(9)
S(2)-S(3)	2.041(9)
S(3)-S(4)	2.052(10)
S(4)-S(5)	2.047(10)
C(2)-C(1)-C(11)	107.4(15)
C(2)-C(1)-S(1)	126.3(15)
C(11)-C(1)-S(1)	126.0(14)
O(1)-C(2)-N(1)	118.6(16)
O(1)-C(2)-C(1)	132.2(17)
N(1)-C(2)-C(1)	109.3(15)
C(8)-C(3)-C(4)	120.8(17)
C(8)-C(3)-N(1)	115.9(16)
C(4)-C(3)-N(1)	123.3(16)
C(5)-C(4)-C(3)	118.4(19)
C(5)-C(4)-H(4)	120.8
C(3)-C(4)-H(4)	120.8
C(6)-C(5)-C(4)	121.5(18)
C(6)-C(5)-C(14)	121.1(18)
C(4)-C(5)-C(14)	117.4(19)
C(7)-C(6)-C(5)	118.6(18)
C(7)-C(6)-C(15)	119.8(18)
C(5)-C(6)-C(15)	121.6(18)
C(6)-C(7)-C(8)	122.1(18)
C(6)-C(7)-H(7)	118.9
C(8)-C(7)-H(7)	118.9
C(3)-C(8)-N(2)	123.7(16)
C(3)-C(8)-C(7)	118.4(17)
N(2)-C(8)-C(7)	117.9(16)
N(2)-C(9)-C(10)	125.2(17)
N(2)-C(9)-C(16)	113.0(17)
C(10)-C(9)-C(16)	121.8(17)
C(11)-C(10)-N(1)	107.7(15)
C(11)-C(10)-C(9)	136.7(17)
N(1)-C(10)-C(9)	115.4(16)
C(10)-C(11)-C(1)	107.2(15)
C(10)-C(11)-S(5)	127.5(14)
C(1)-C(11)-S(5)	125.3(14)
O(1)-C(12)-C(13)	109.4(18)
O(1)-C(12)-H(12A)	109.8
C(13)-C(12)-H(12A)	109.8
O(1)-C(12)-H(12B)	109.8
C(13)-C(12)-H(12B)	109.8
H(12A)-C(12)-H(12B)	108.2
C(12)-C(13)-H(13A)	109.5
C(12)-C(13)-H(13B)	109.5
H(13A)-C(13)-H(13B)	109.5
C(12)-C(13)-H(13C)	109.5
H(13A)-C(13)-H(13C)	109.5
H(13B)-C(13)-H(13C)	109.5
C(5)-C(14)-H(14A)	109.5
C(5)-C(14)-H(14B)	109.5

H(14A)-C(14)-H(14B)	109.5
C(5)-C(14)-H(14C)	109.5
H(14A)-C(14)-H(14C)	109.5
H(14B)-C(14)-H(14C)	109.5
C(6)-C(15)-H(15A)	109.5
C(6)-C(15)-H(15B)	109.5
H(15A)-C(15)-H(15B)	109.5
C(6)-C(15)-H(15C)	109.5
H(15A)-C(15)-H(15C)	109.5
H(15B)-C(15)-H(15C)	109.5
F(2)-C(16)-F(3)	107.9(17)
F(2)-C(16)-F(1)	107.1(17)
F(3)-C(16)-F(1)	106.0(19)
F(2)-C(16)-C(9)	112.0(19)
F(3)-C(16)-C(9)	111.4(17)
F(1)-C(16)-C(9)	112.0(16)
C(2)-N(1)-C(10)	108.3(14)
C(2)-N(1)-C(3)	130.1(14)
C(10)-N(1)-C(3)	121.5(14)
C(9)-N(2)-C(8)	117.5(16)
C(2)-O(1)-C(12)	118.6(15)
C(1)-S(1)-S(2)	102.9(7)
S(3)-S(2)-S(1)	104.3(4)
S(2)-S(3)-S(4)	104.3(4)
S(5)-S(4)-S(3)	104.3(5)
C(11)-S(5)-S(4)	103.7(7)

Table 4. Anisotropic displacement parameters (A^2 x 10^3) for siv-01-50a.
The anisotropic displacement factor exponent takes the form:
-2 pi^2 [h^2 a*^2 U11 + ... + 2 h k a* b* U12]

	U11	U22	U33	U23	U13	U12
C(1)	43(10)	44(10)	59(14)	6(9)	18(10)	3(9)
C(2)	50(11)	28(9)	70(15)	5(9)	23(12)	-2(8)
C(3)	38(10)	42(10)	48(13)	2(9)	12(10)	-2(8)
C(4)	49(12)	36(10)	66(15)	3(10)	11(12)	3(8)
C(5)	50(11)	42(10)	66(15)	-1(10)	11(11)	8(9)
C(6)	40(10)	51(11)	63(15)	-5(11)	9(11)	0(9)
C(7)	42(10)	46(11)	67(15)	-2(10)	15(11)	-4(8)
C(8)	51(11)	31(9)	61(14)	2(9)	22(10)	-2(8)
C(9)	42(10)	35(9)	58(13)	2(10)	8(11)	0(8)
C(10)	44(11)	37(10)	49(13)	2(9)	11(10)	2(8)
C(11)	39(10)	44(10)	53(13)	-4(9)	12(10)	8(8)
C(12)	48(12)	47(12)	100(20)	9(12)	13(14)	-13(9)
C(13)	100(20)	60(15)	120(30)	2(15)	10(20)	-25(14)
C(14)	72(16)	49(12)	90(20)	-7(12)	21(16)	16(11)
C(15)	53(14)	84(17)	85(19)	9(14)	33(15)	6(11)
C(16)	64(14)	40(10)	63(16)	-5(11)	13(13)	-6(10)
F(1)	64(8)	53(7)	80(9)	2(6)	9(8)	11(6)
F(2)	79(9)	53(7)	95(11)	-22(8)	4(8)	-7(7)
F(3)	90(10)	43(7)	109(12)	8(7)	47(9)	-7(6)
N(1)	41(8)	34(8)	54(11)	3(7)	17(8)	0(6)
N(2)	45(9)	38(8)	64(12)	2(7)	23(9)	-1(7)
O(1)	51(8)	37(7)	75(11)	9(7)	19(8)	0(6)

S(1)	54(3)	64(3)	58(4)	7(3)	24(3)	2(2)
S(2)	45(3)	79(4)	109(6)	-2(4)	16(4)	-1(3)
S(3)	54(3)	83(4)	130(7)	-3(4)	29(4)	16(3)
S(4)	67(4)	65(4)	105(6)	7(4)	17(4)	20(3)
S(5)	59(3)	51(3)	72(4)	-16(3)	22(3)	2(2)

Table 5. Hydrogen bonds for siv0150a [A and deg.].

Donor --- H....Acceptor	D - H	H...A	D...A	D - H...A
C(4) --H(4) ..O(1)	0.93	2.23	2.84(3)	122
C(12) --H(12B) ..S(1)	0.97	2.81	3.34(2)	115

Compounds 6-8

General

The compounds **6**–**8** were synthesized by the method of Amelichev with modifications [2].

Scheme S2. Synthetic route to **6-8.**

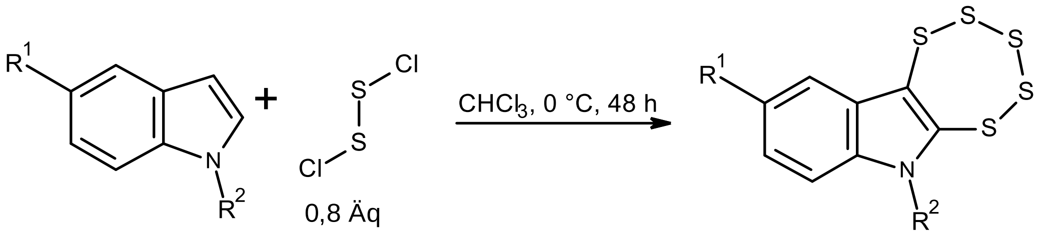

compound	R^1	R^2
**6**	H	CH_3
**7**	Cl	CH_2-CH_3
**8**	H	CH_2-C_6H_5

NMR

Bruker Ft-NMR-Spektrometer Avance II Ultrashield 400 MHz. Temperature 25 °C; ^{1}H-Spectra: 400.20 MHz, ^{13}C-, DEPT135-, HSQC, HMBC Spectra: 100.61 MHz; internal standard: Tetramethylsilane (TMS)

RP-HPLC

RP-HPLC were performed with a Merck-Hitachi LaChrom 7000 instrument fitted with a Merck Chromolith SpeedROD RP-18e column (4.6 x 50 mm) held at 30 °C. Samples of 25 µL were injected and eluted with a solvent of 80% acetonitrile/water at a flow rate of 1.0 mL/min for purity testing and 1.25 mL/min for stability testing (**4**). Detection was done between the wavelengths between 210 and 500 nm.

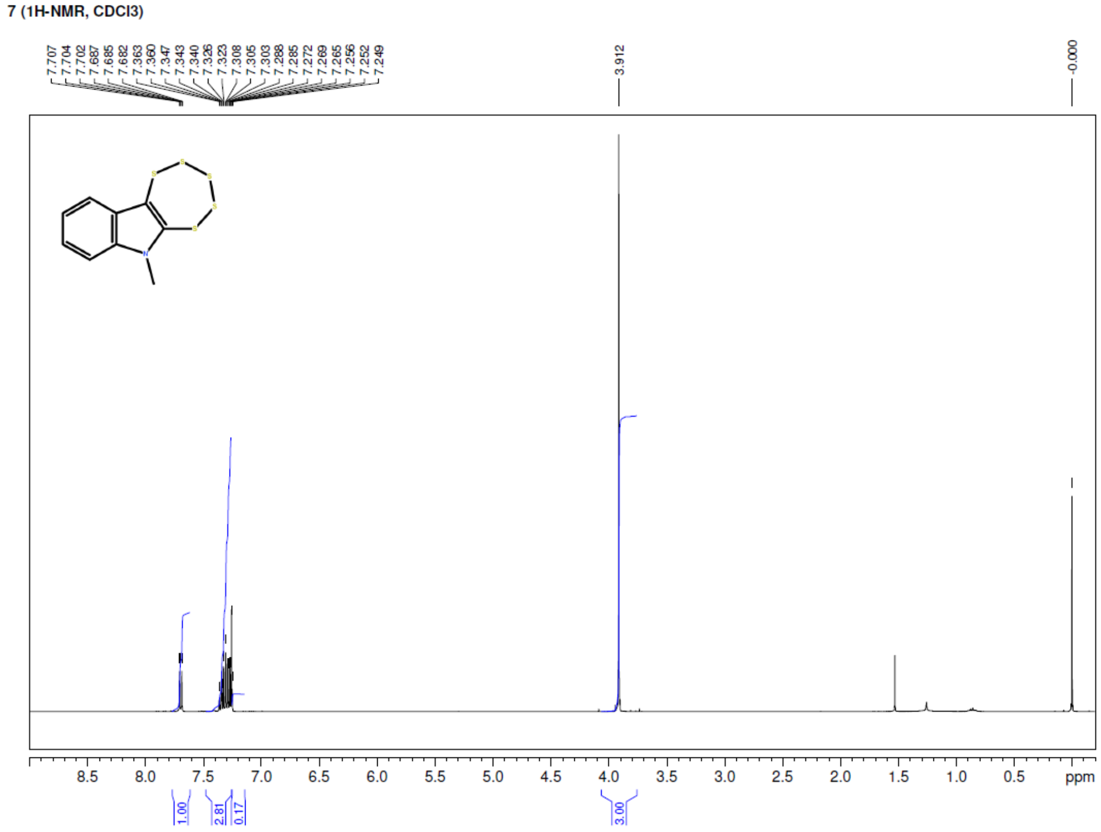

Figure S18. ^{1}H-NMR ($CDCl_3$) of compound **6**.

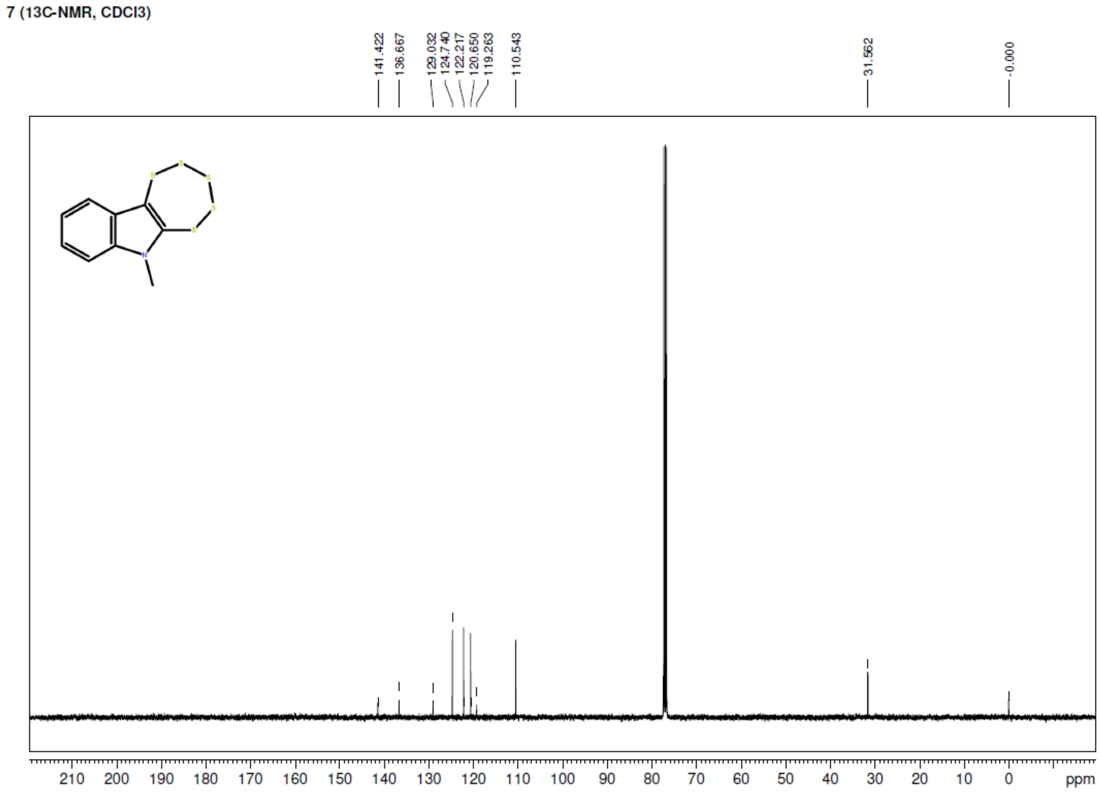

Figure S19. ^{13}C-NMR ($CDCl_3$) of compound **6**.

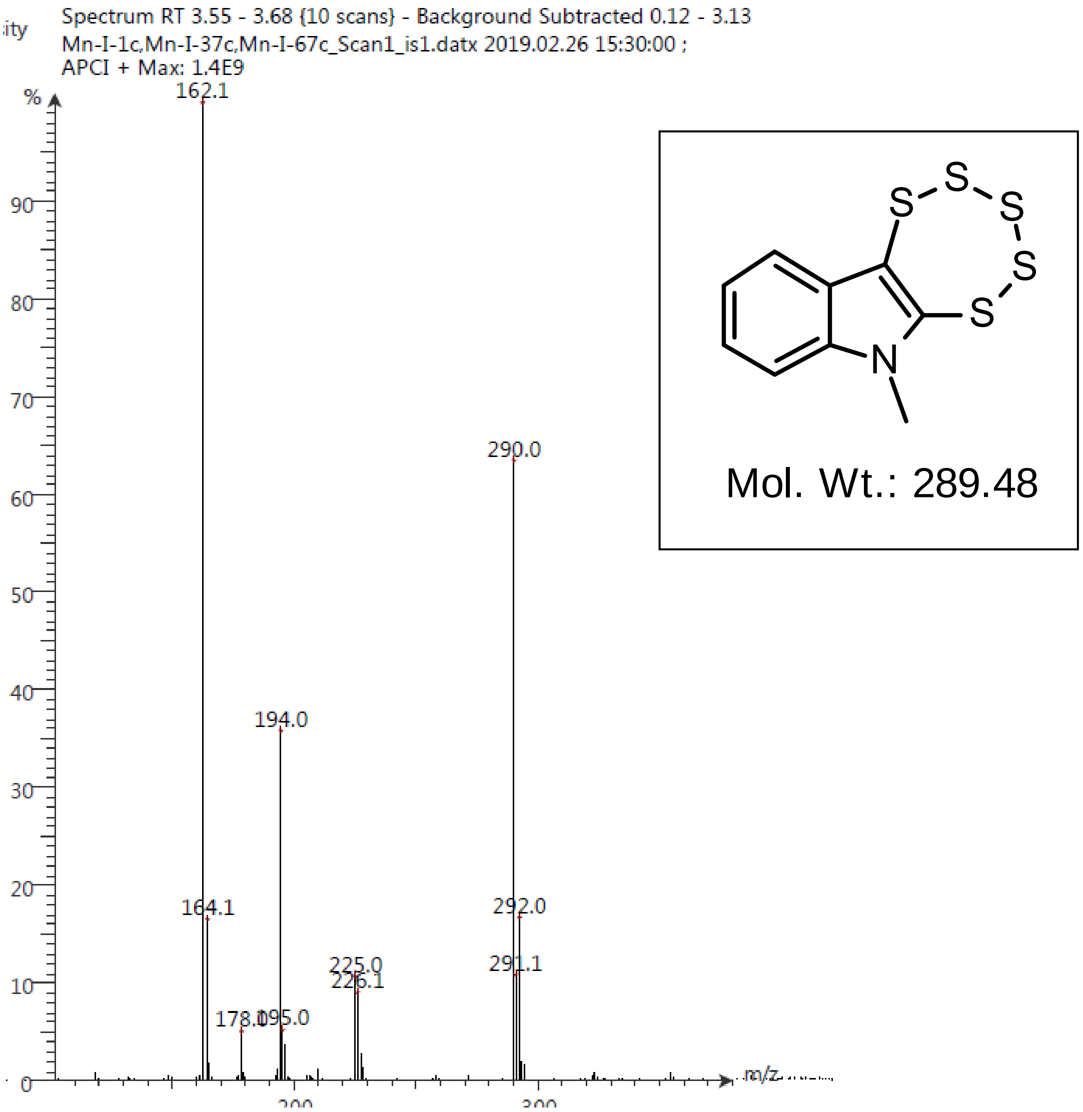

Figure S20. APCI-MS of compound **6**.

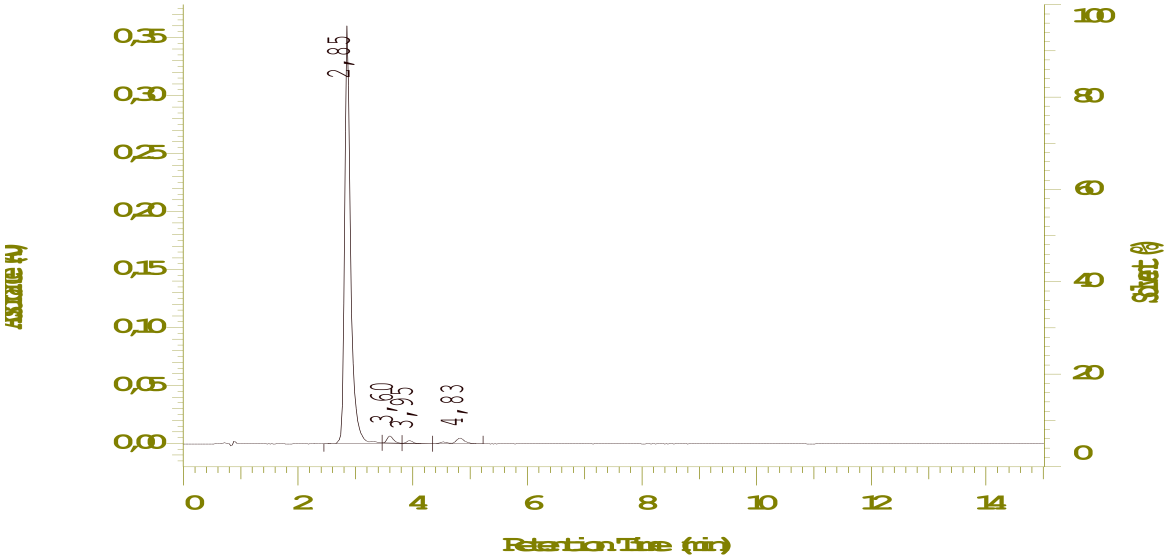

Figure S21. HPLC of compound **6**, rt = 2.85 min, detected at λ = 250 nm.

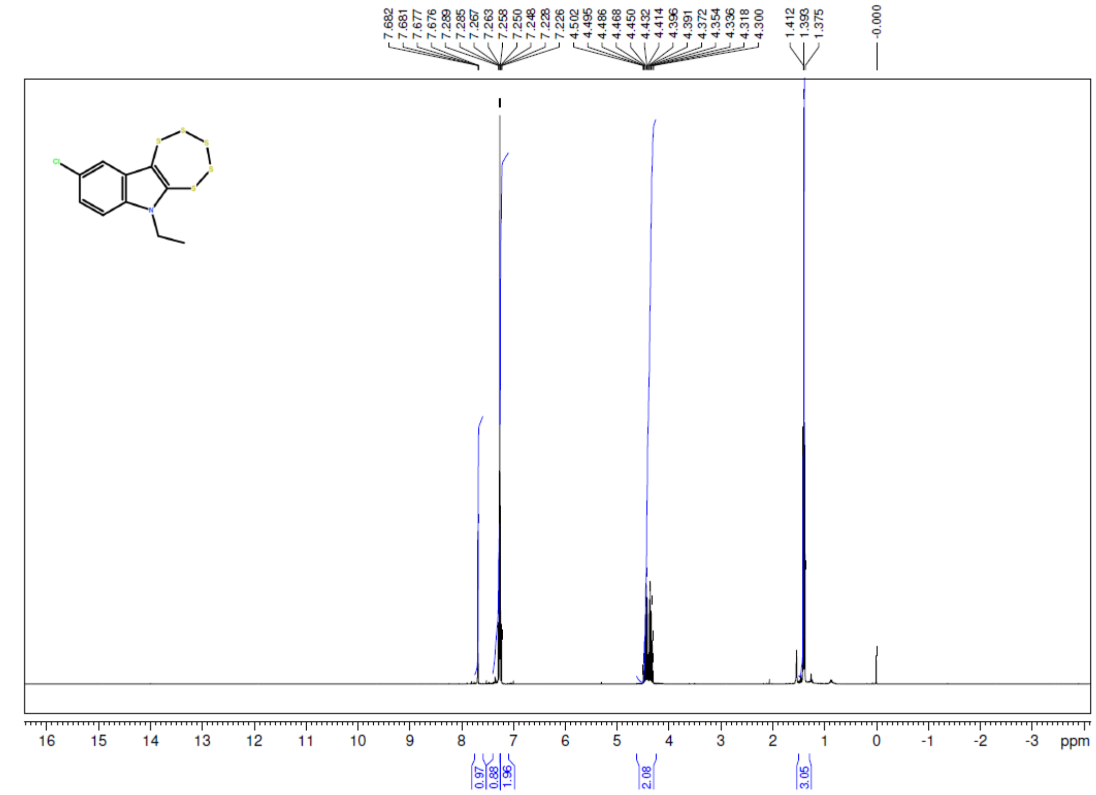

Figure S22. ^{1}H-NMR ($CDCl_3$) of compound **7**.

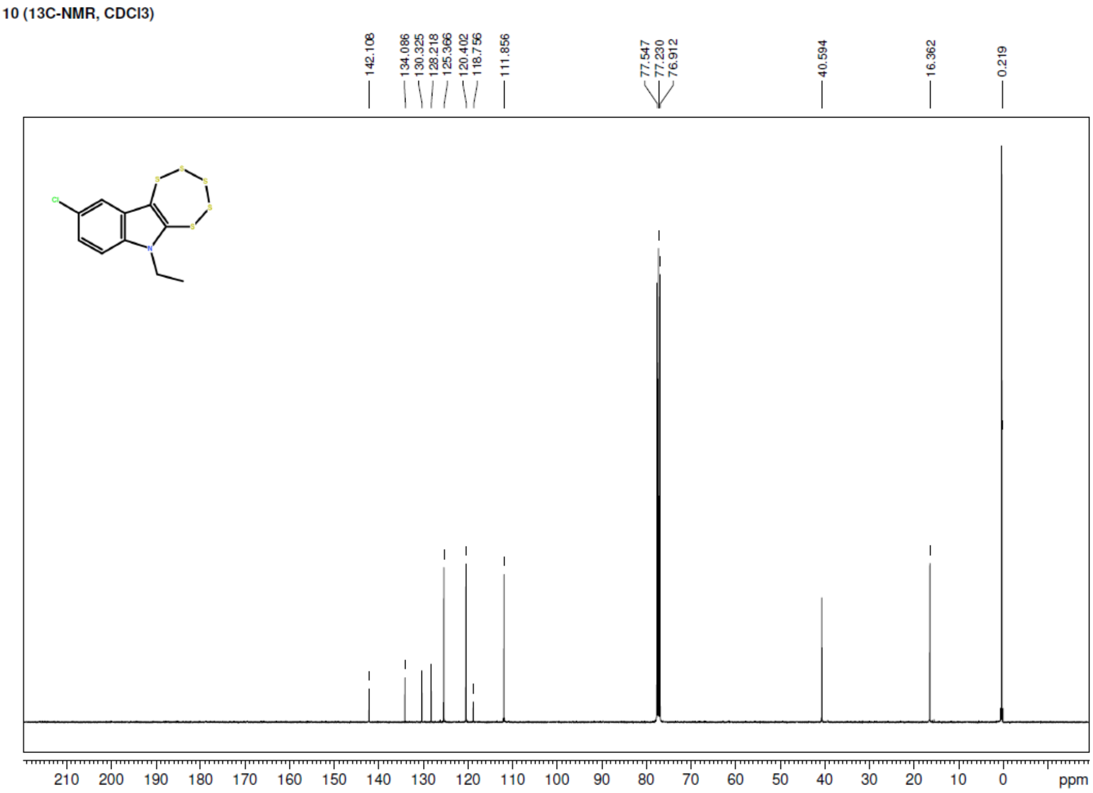

Figure S23. ^{13}C-NMR ($CDCl_3$) of compound **7**.

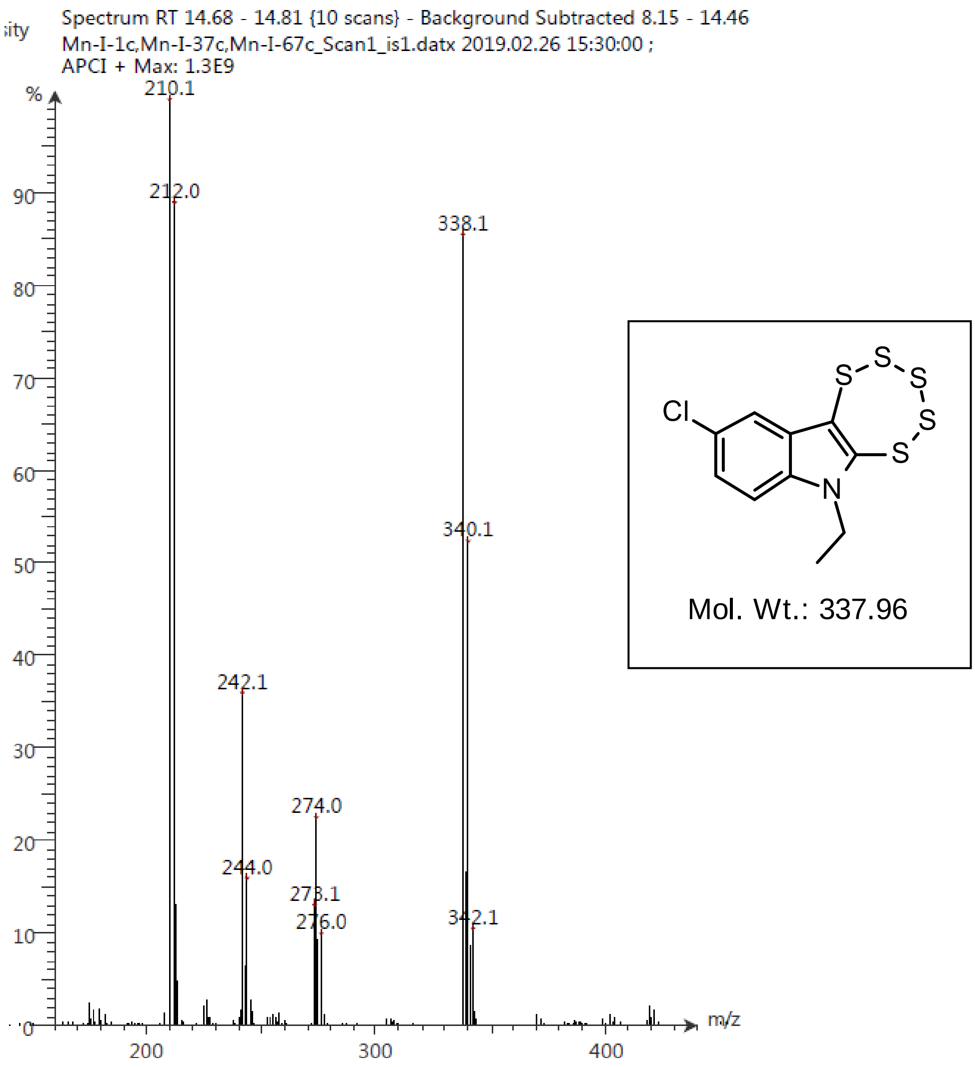

Figure S24. APCI-MS of **7**.

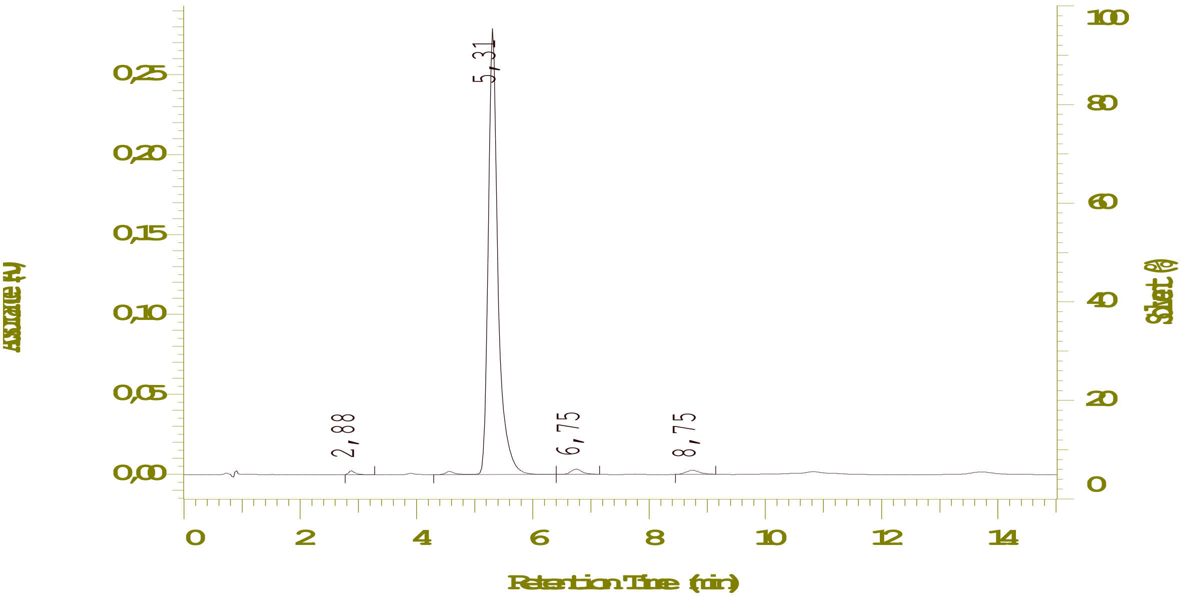

Figure S25. RP-HPLC of compound **7**, rt= 5.31 min, detected at λ = 250 nm.

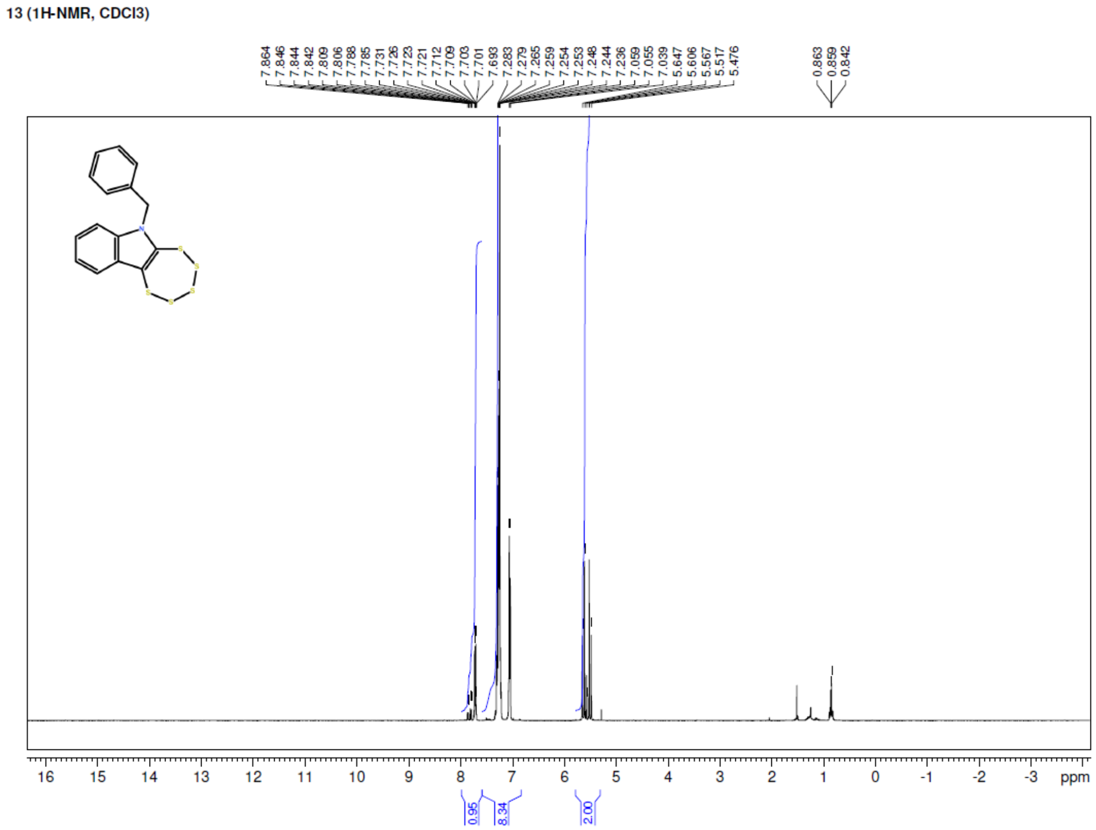

Figure S26. ^{1}H-NMR ($CDCl_3$) of compound **8**.

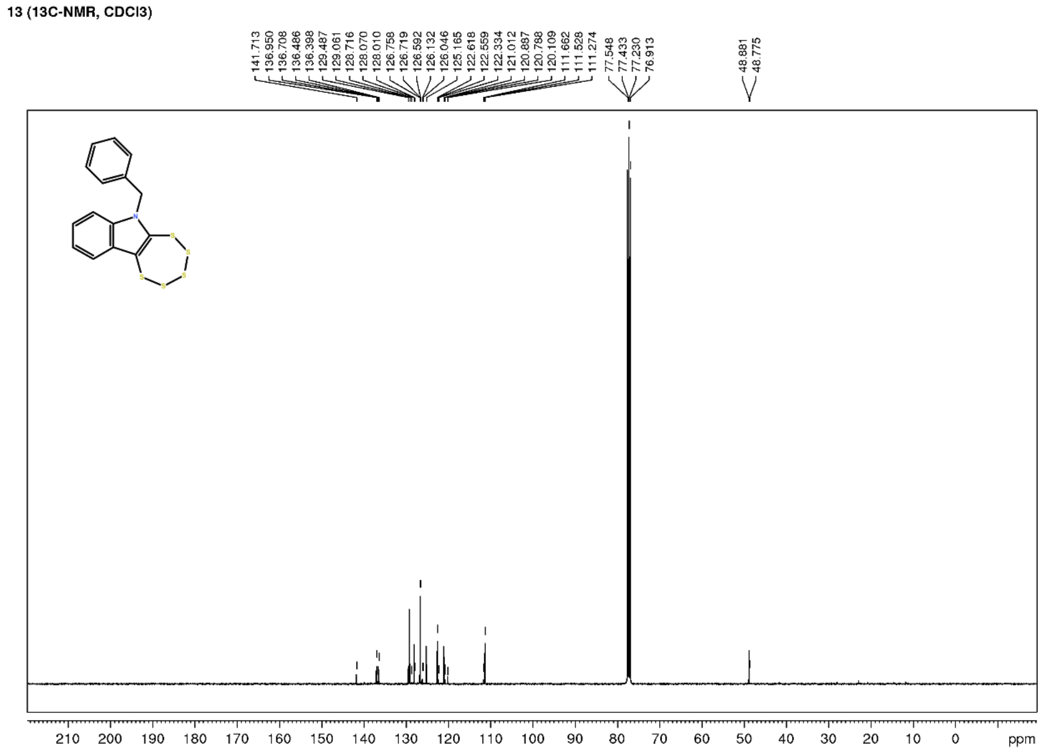

Figure S27. ^{13}C-NMR ($CDCl_3$) of compound **8**.

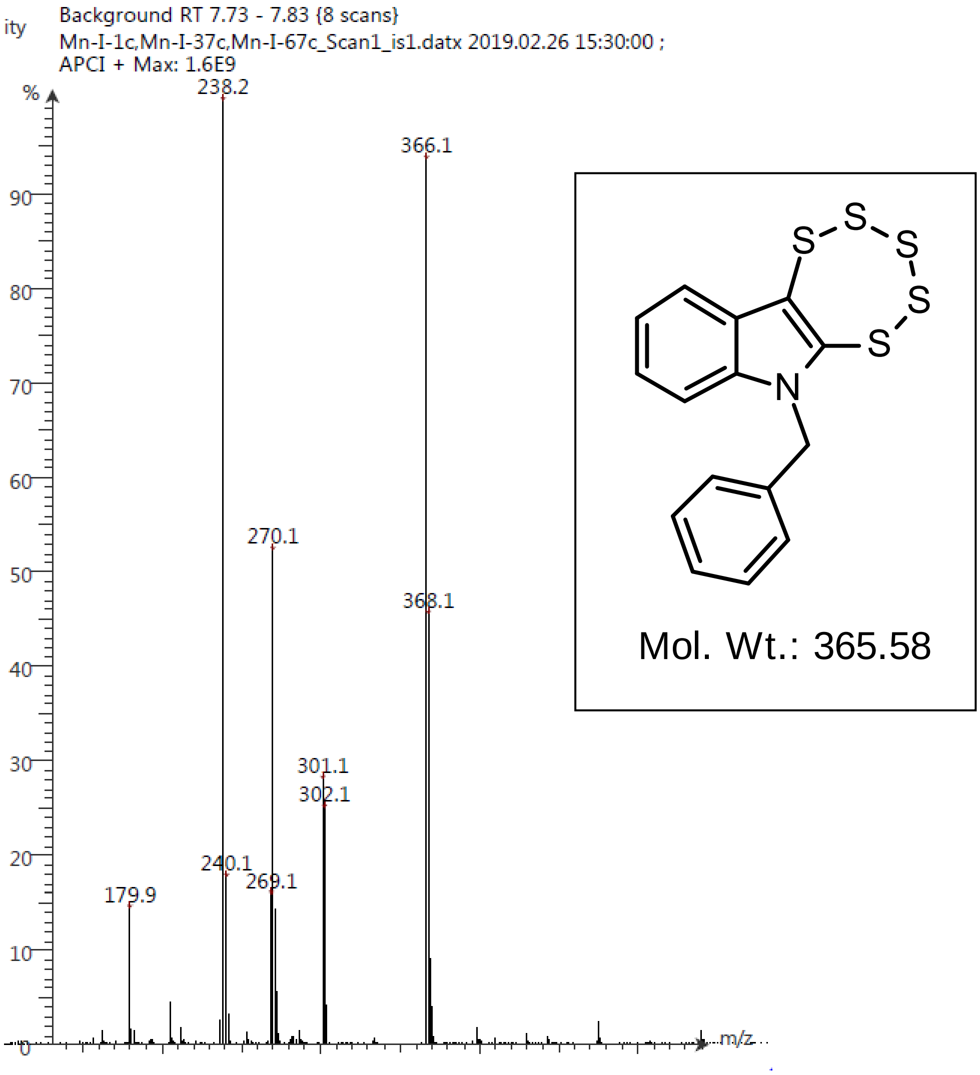

Figure S28. APCI-MS of main peak in **8**.

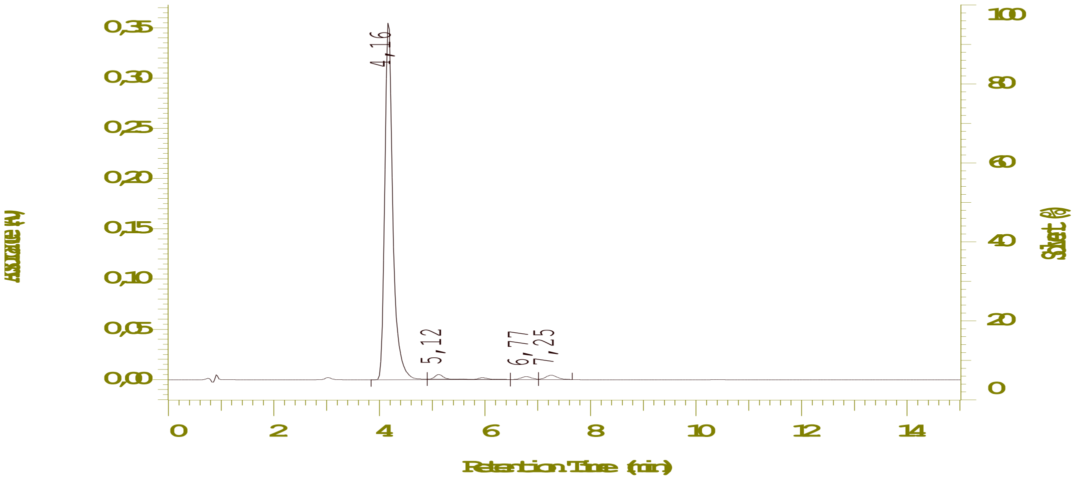

Figure S29. RP-HPLC of compound **8**, rt = 4.16 min, detected at λ = 250 nm.

X-ray Crystallographic data for 8

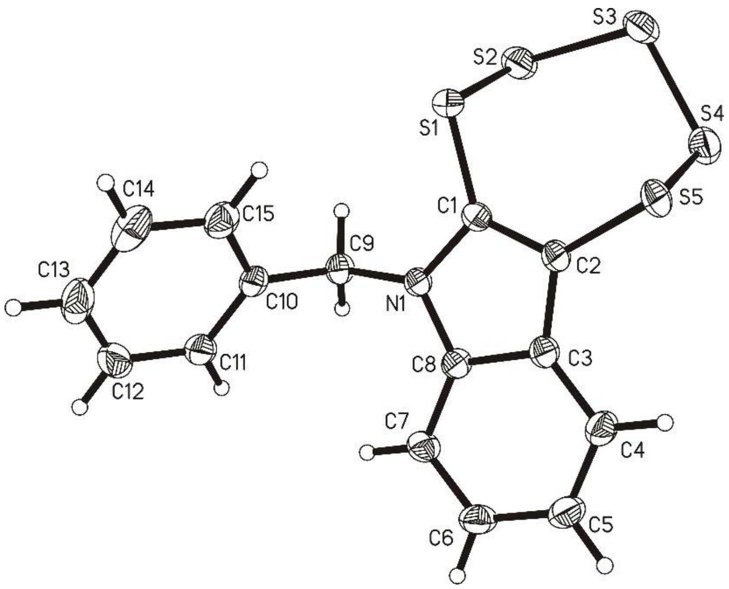

Figure S30. X-Ray crystal structure for compound **8**

Table S2. Crystal data and structure refinement for **8**.

Identification code	MN-I-37c	
Empirical formula	$C_{15}H_{11}N_1S_5$	
Formula weight	365.55	
Temperature	170(2) K	
Wavelength	0.71073 A	
Crystal system, space group	Triclinic, *P*-1	
Unit cell dimensions	a = 8.7059(17) A	alpha = 108.20(3)°
	b = 9.768(2) A	beta = 102.15(3)°
	c = 10.084(2) A	gamma = 102.42(3)°
Volume	759.2(3) $Å^3$	
Z, Calculated density	2, 1.599 g/cm^3	
Absorption coefficient	0.753 mm^-1	
F(000)	376	
Crystal size	0.424 x 0.165 x 0.085 mm	
Theta range for data collection	3.589° to 29.198°	
Limiting indices	-11<=h<=11, -12<=k<=13, -13<=l<=13	
Reflections collected / unique	8819 / 4049 [R(int) = 0.0358]	

Completeness to theta = 25.242	99.3 %
Absorption correction	Numerical
Max. and min. transmission	0.9846 and 0.9200
Refinement method	Full-matrix least-squares on F^2
Data / restraints / parameters	4049 / 0 / 190
Goodness-of-fit on F^2	1.050
Final R indices [I>2sigma(I)]	R1 = 0.0389, wR2 = 0.0979
R indices (all data)	R1 = 0.0562, wR2 = 0.1051
Extinction coefficient	n/a
Largest diff. peak and hole	0.859 and -0.496 e·$Å^{-3}$

Table 2. Atomic coordinates (x 10^4) and equivalent isotropic displacement parameters (A^2 x 10^3) for **9**. U(eq) is defined as one third of the trace of the orthogonalized Uij tensor.

	x	y	z	U(eq)
C(1)	4628(2)	6856(3)	3350(2)	23(1)
C(2)	5711(2)	7728(3)	4719(2)	24(1)
C(3)	4809(2)	8467(2)	5579(2)	23(1)
C(4)	5215(3)	9454(3)	7034(2)	28(1)
C(5)	3994(3)	9954(3)	7519(2)	31(1)
C(6)	2382(3)	9503(3)	6580(3)	30(1)
C(7)	1948(2)	8532(3)	5146(2)	26(1)
C(8)	3180(2)	8010(2)	4660(2)	22(1)
C(9)	1533(2)	6095(2)	2160(2)	23(1)
C(10)	868(2)	6890(2)	1214(2)	23(1)
C(11)	-609(3)	7213(3)	1243(2)	30(1)
C(12)	-1292(3)	7850(3)	312(3)	39(1)
C(13)	-515(4)	8173(3)	-661(3)	43(1)
C(14)	959(4)	7872(3)	-692(3)	39(1)
C(15)	1648(3)	7233(3)	244(2)	30(1)
N(1)	3080(2)	7010(2)	3311(2)	22(1)
S(1)	5039(1)	5778(1)	1807(1)	29(1)
S(2)	5316(1)	3899(1)	2238(1)	33(1)
S(3)	7746(1)	4533(1)	3430(1)	33(1)
S(4)	7874(1)	5910(1)	5484(1)	33(1)
S(5)	7806(1)	7938(1)	5283(1)	29(1)

Table 3. Bond lengths [A] and angles [deg] for **13**.

C(1)-C(2)	1.376(3)
C(1)-N(1)	1.381(2)
C(1)-S(1)	1.741(2)
C(2)-C(3)	1.425(3)
C(2)-S(5)	1.738(2)
C(3)-C(4)	1.399(3)
C(3)-C(8)	1.409(3)

C(4)-C(5)	1.379(3)
C(5)-C(6)	1.405(3)
C(6)-C(7)	1.376(3)
C(7)-C(8)	1.397(3)
C(8)-N(1)	1.377(3)
C(9)-N(1)	1.461(3)
C(9)-C(10)	1.508(3)
C(10)-C(15)	1.385(3)
C(10)-C(11)	1.392(3)
C(11)-C(12)	1.383(4)
C(12)-C(13)	1.378(4)
C(13)-C(14)	1.381(4)
C(14)-C(15)	1.390(4)
S(1)-S(2)	2.0645(10)
S(2)-S(3)	2.0505(12)
S(3)-S(4)	2.0520(12)
S(4)-S(5)	2.0652(10)
C(2)-C(1)-N(1)	109.55(18)
C(2)-C(1)-S(1)	128.38(16)
N(1)-C(1)-S(1)	121.98(15)
C(1)-C(2)-C(3)	107.28(17)
C(1)-C(2)-S(5)	126.46(16)
C(3)-C(2)-S(5)	126.23(16)
C(4)-C(3)-C(8)	119.59(19)
C(4)-C(3)-C(2)	133.96(19)
C(8)-C(3)-C(2)	106.44(18)
C(5)-C(4)-C(3)	118.4(2)
C(4)-C(5)-C(6)	121.3(2)
C(7)-C(6)-C(5)	121.6(2)
C(6)-C(7)-C(8)	117.2(2)
N(1)-C(8)-C(7)	129.36(18)
N(1)-C(8)-C(3)	108.60(17)
C(7)-C(8)-C(3)	122.03(19)
N(1)-C(9)-C(10)	114.39(18)
C(15)-C(10)-C(11)	118.7(2)
C(15)-C(10)-C(9)	121.22(19)
C(11)-C(10)-C(9)	119.9(2)
C(12)-C(11)-C(10)	120.6(2)
C(13)-C(12)-C(11)	120.2(2)
C(12)-C(13)-C(14)	119.8(2)
C(13)-C(14)-C(15)	120.1(3)
C(10)-C(15)-C(14)	120.5(2)
C(8)-N(1)-C(1)	108.10(17)
C(8)-N(1)-C(9)	124.12(16)
C(1)-N(1)-C(9)	126.95(18)
C(1)-S(1)-S(2)	103.43(8)
S(3)-S(2)-S(1)	104.34(5)
S(2)-S(3)-S(4)	104.81(5)
S(3)-S(4)-S(5)	104.24(4)
C(2)-S(5)-S(4)	103.61(9)

Symmetry transformations used to generate equivalent atoms:

Table 4. Anisotropic displacement parameters (A^2 x 10^3) for **8**. The anisotropic displacement factor exponent takes the form: -2 pi^2 [h^2 a*^2 U11 + ... + 2 h k a* b* U12]

	U11	U22	U33	U23	U13	U12
C(1)	17(1)	30(1)	22(1)	8(1)	7(1)	8(1)
C(2)	14(1)	32(1)	22(1)	7(1)	4(1)	6(1)
C(3)	17(1)	26(1)	23(1)	7(1)	5(1)	4(1)
C(4)	23(1)	30(1)	23(1)	3(1)	3(1)	4(1)
C(5)	32(1)	30(1)	24(1)	2(1)	8(1)	8(1)
C(6)	28(1)	31(1)	29(1)	6(1)	13(1)	11(1)
C(7)	18(1)	29(1)	28(1)	8(1)	9(1)	7(1)
C(8)	17(1)	24(1)	21(1)	7(1)	6(1)	3(1)
C(9)	16(1)	25(1)	22(1)	4(1)	2(1)	2(1)
C(10)	19(1)	22(1)	18(1)	1(1)	0(1)	2(1)
C(11)	24(1)	35(1)	24(1)	3(1)	3(1)	11(1)
C(12)	38(1)	36(1)	31(1)	1(1)	-1(1)	20(1)
C(13)	62(2)	28(1)	25(1)	3(1)	-6(1)	18(1)
C(14)	53(2)	29(1)	24(1)	8(1)	5(1)	3(1)
C(15)	26(1)	32(1)	23(1)	5(1)	4(1)	4(1)
N(1)	14(1)	29(1)	19(1)	5(1)	4(1)	5(1)
S(1)	26(1)	41(1)	21(1)	9(1)	9(1)	16(1)
S(2)	26(1)	34(1)	35(1)	8(1)	10(1)	11(1)
S(3)	26(1)	42(1)	38(1)	17(1)	14(1)	17(1)
S(4)	28(1)	46(1)	31(1)	19(1)	9(1)	16(1)
S(5)	14(1)	38(1)	30(1)	10(1)	3(1)	6(1)

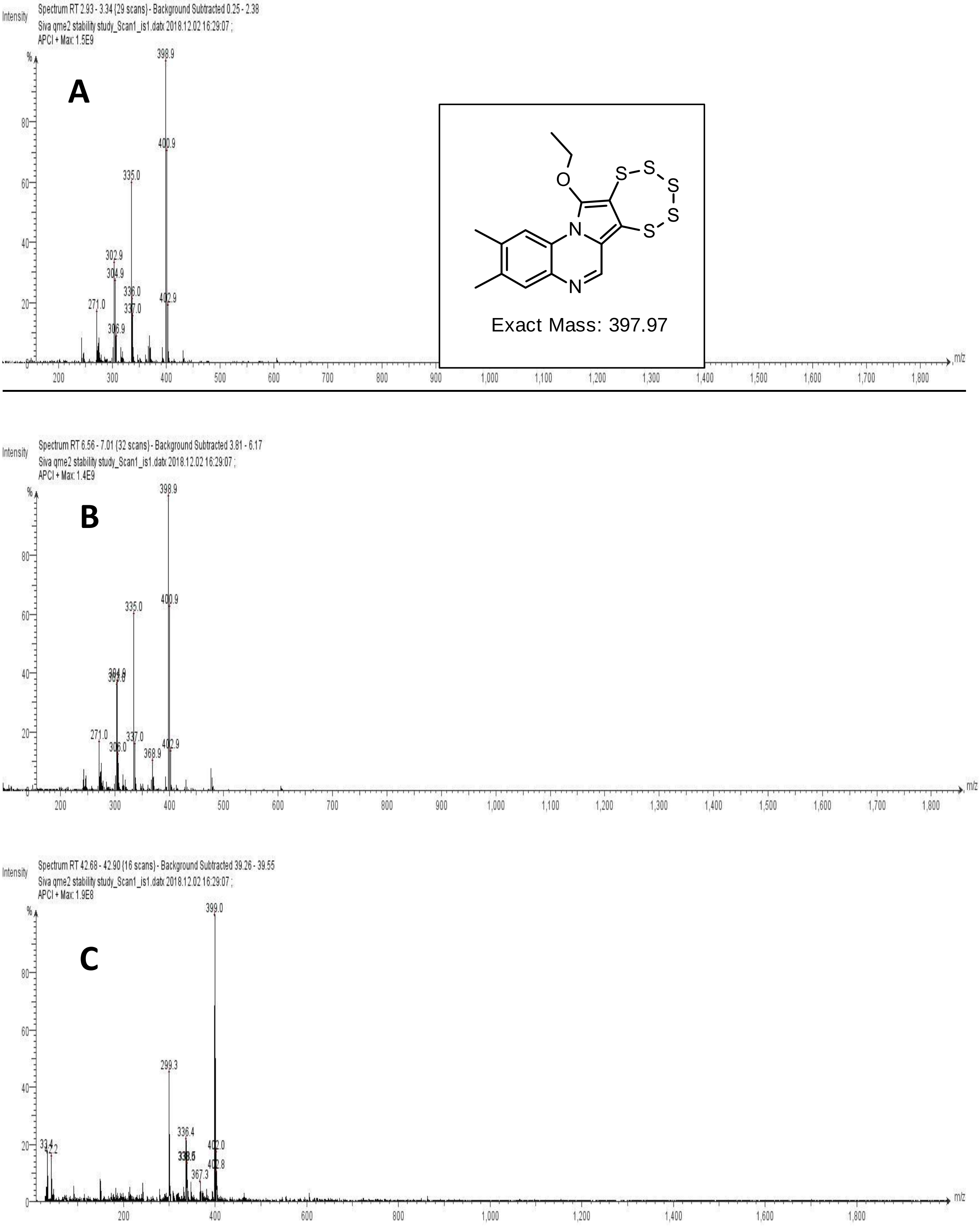

Figure S31. APCI-MS of **4** in **A**) in $CHCl_3$, **B**) in 1:1 DMSO/water solution of 10 mM GSH at T= 0, **C**) in 1:1 DMSO/water solution of 10 mM GSH at T = 60 min at 23 °C.

A

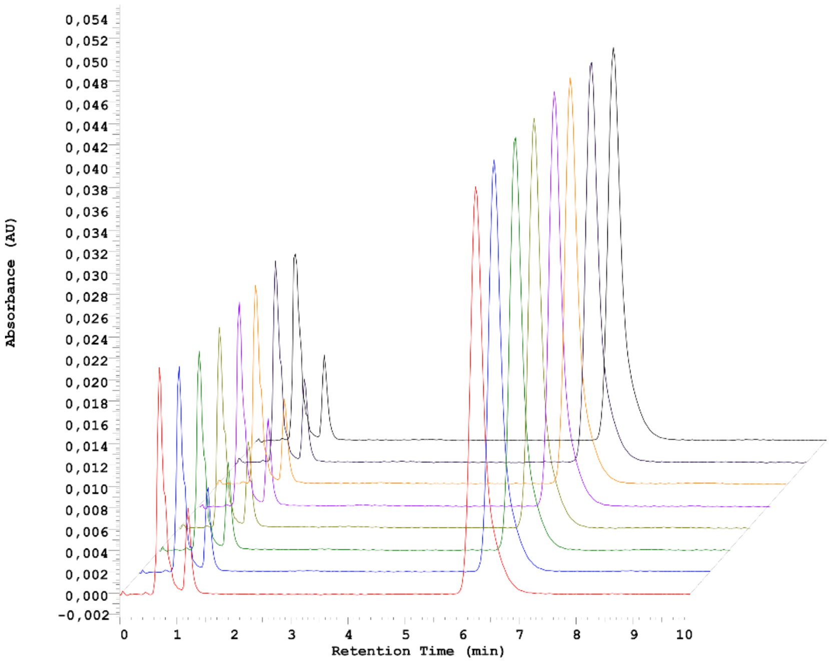
Absorbance (AU)
0,054
0,052
0,050
0,048
0,046
0,044
0,042
0,040
0,038
0,036
0,034
0,032
0,030
0,028
0,026
0,024
0,022
0,020
0,018
0,016
0,014
0,012
0,010
0,008
0,006
0,004
0,002
0,000
-0,002
0 1 2 3 4 5 6 7 8 9 10
Retention Time (min)

B

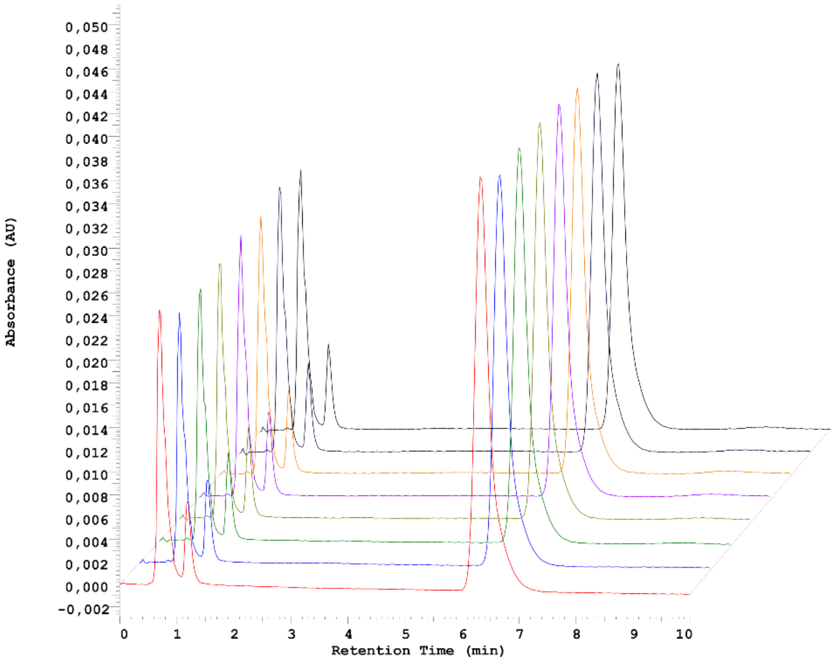
Absorbance (AU)
0,050
0,048
0,046
0,044
0,042
0,040
0,038
0,036
0,034
0,032
0,030
0,028
0,026
0,024
0,022
0,020
0,018
0,016
0,014
0,012
0,010
0,008
0,006
0,004
0,002
0,000
-0,002
0 1 2 3 4 5 6 7 8 9 10
Retention Time (min)

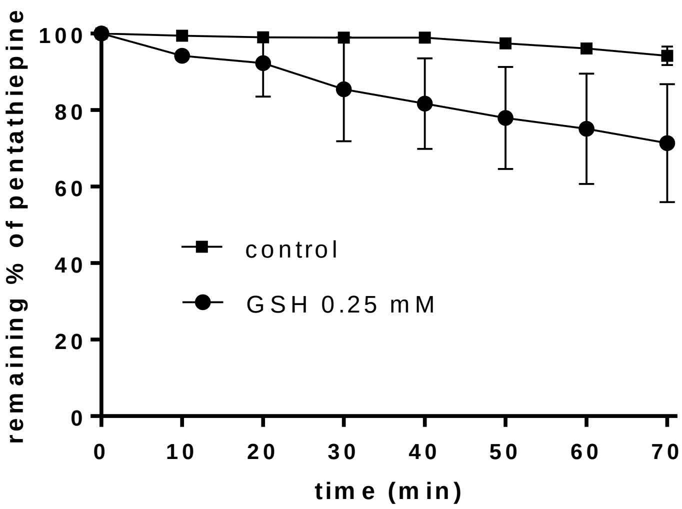

Figure S32. Stability of **4** (20 µM) in buffer used for the GPx assay **a**) without and **b**) with 250 µM GSH at 23 °C. Figure shows representative RP-HPLC chromatograms recorded every 10 min over 70 min. The peak at 6.2 min is from **5**, peaks at 0.7 and 1.2 min are from the buffer. **c**) Stability curves of data from **a** and **b**. Data points are averages and error bars are standard deviations of 3 independent experiments.

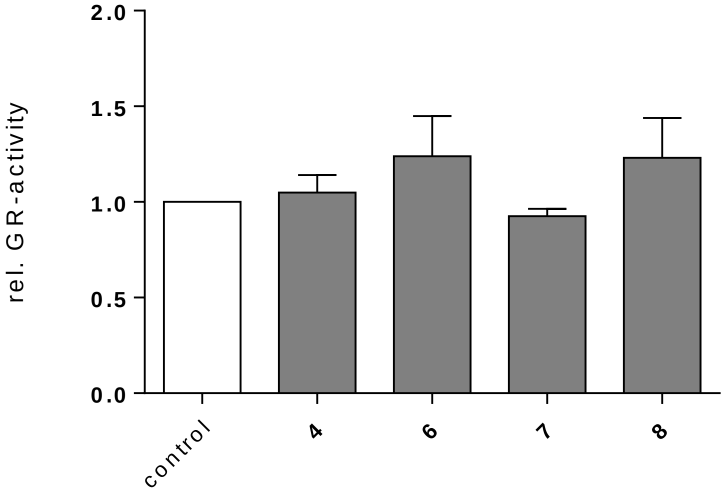

Figure S33. Relative GR activity after incubation with various pentathiepins 20 µM at 23 °C (mean + SD; n=3).

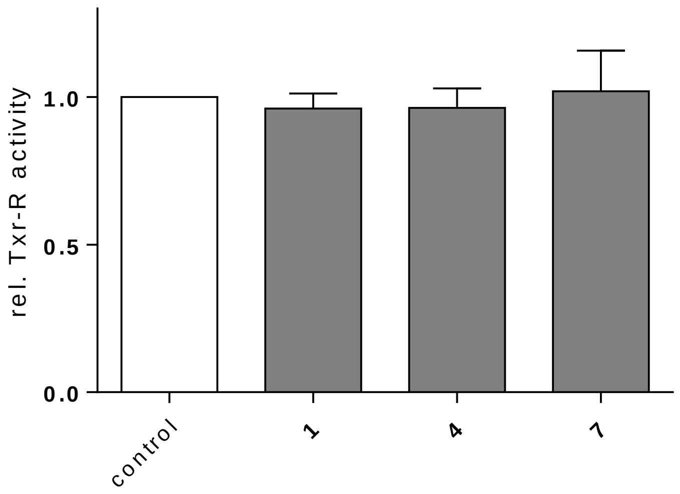

Figure S34. Relative Trx-R activity after incubation with 25 µM **1**, **4** and **7** at 23 °C (mean + SD; n=3).

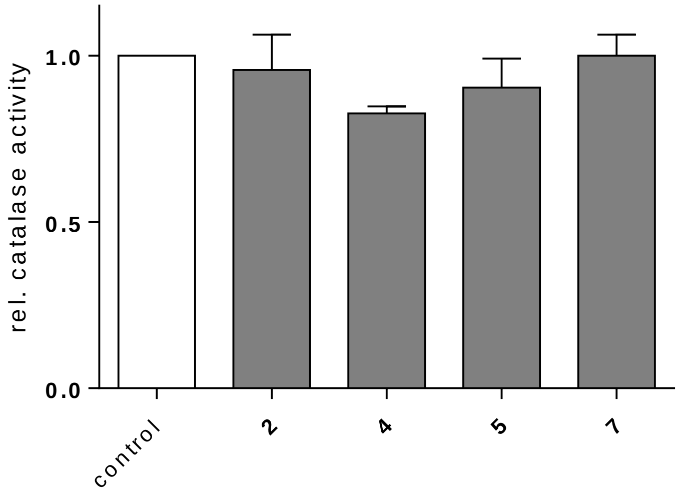

Figure S35. Relative CAT activity after incubation with pentathiepins 25 µM at 23 °C (mean + SD; n=3).

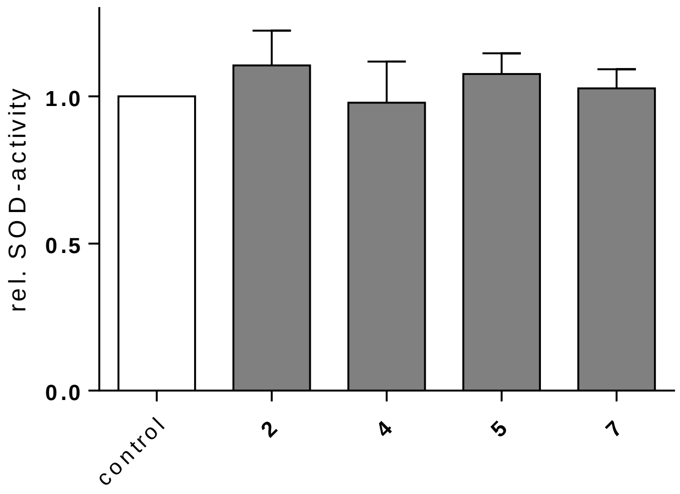

Figure S36. Relative SOD activity after incubation with pentathiepins 25 µM at 23 °C (mean + SD; n=3).

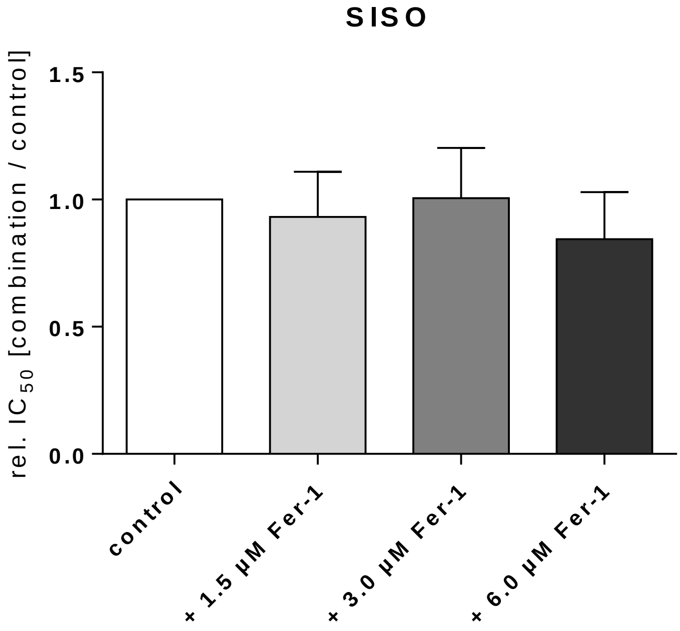

Figure S37. Relative IC_{50} value of **4** in the presence or absence of ferrostatin-1. IC_{50} values were determined via MTT-viability assay after 48 h incubation time [mean + SD; n=3].

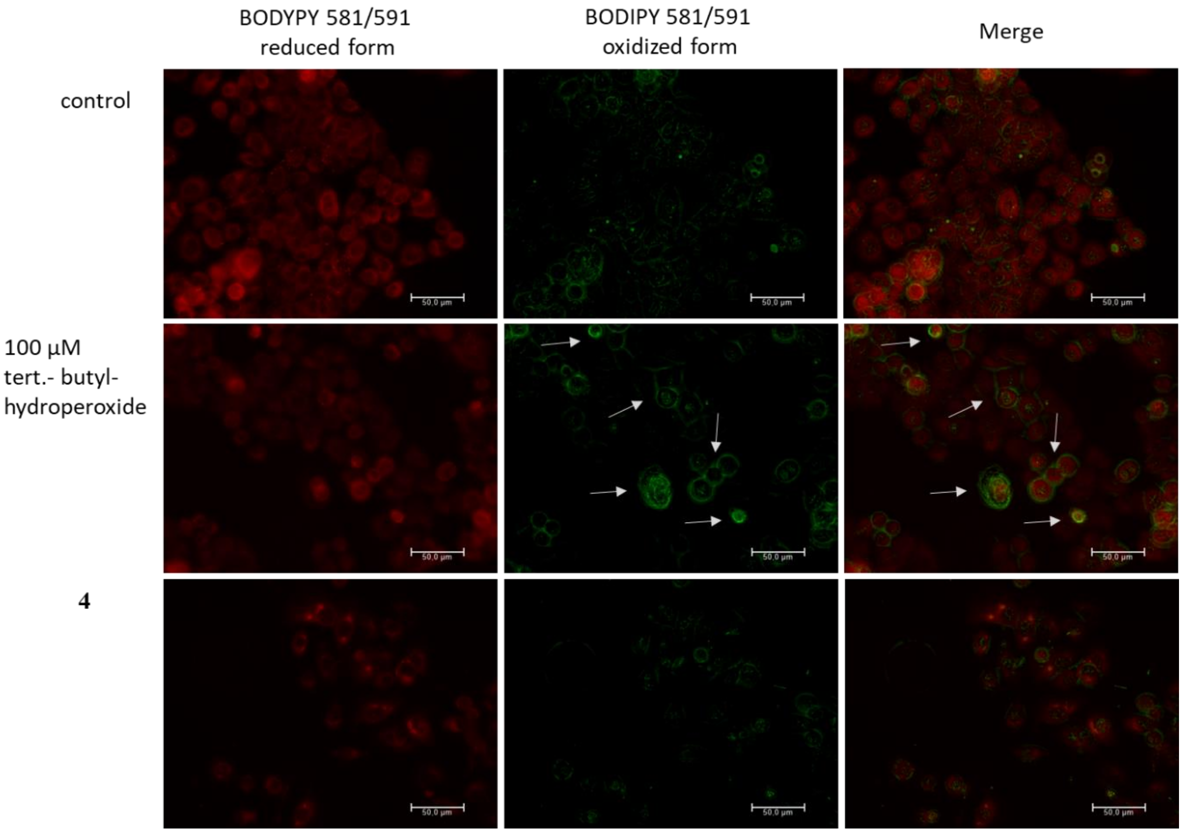

Figure S38. Fluorescence microscopy of SISO cells after incubation with 25 µM **4** or 100 µM tert. butyl-hydroperoxide for 24 h, followed by staining with BODIPY 581/591 dye; Left column: reduced BODIPY form, emission of red light (TXR-channel); middle column: evidence of LPO by visualization of oxidized BODIPY highlighted by arrows, emission of green light (FITC-channel); right column: overlay.

References:

[1] Zubair, M., A.C. Ghosh, and C. Schulzke, The unexpected and facile molybdenum mediated formation of tri- and tetracyclic pentathiepins from pyrazine-alkynes and sulfur. Chemical Communications, 2013. 49(39): p. 4343-4345.

[2] Amelichev, S.A., et al., *Direct synthesis of fused 1,2,3,4,5-pentathiepins.* Org Biomol Chem, 2005. **3**(19): p. 3496-501.
